# Chronic hepatitis C viral infection subverts vaccine‐induced T‐cell immunity in humans

**DOI:** 10.1002/hep.28294

**Published:** 2016-01-22

**Authors:** Christabel Kelly, Leo Swadling, Stefania Capone, Anthony Brown, Rachel Richardson, John Halliday, Annette von Delft, Ye Oo, David Mutimer, Ayako Kurioka, Felicity Hartnell, Jane Collier, Virginia Ammendola, Mariarosaria Del Sorbo, Fabiana Grazioli, Maria Luisa Esposito, Stefania Di Marco, Loredana Siani, Cinzia Traboni, Adrian V.S. Hill, Stefano Colloca, Alfredo Nicosia, Riccardo Cortese, Antonella Folgori, Paul Klenerman, Eleanor Barnes

**Affiliations:** ^1^ Nuffield Department of MedicineUniversity of OxfordOxfordUK; ^2^ Oxford NIHR BRC and Translational Gastroenterology UnitOxfordUK; ^3^ ReiThera Srl (formerly Okairos Srl)Viale Città d'EuropaRomeItaly; ^4^ Department of HepatologyQueen Elizabeth HospitalBirminghamUK; ^5^ The Jenner InstituteUniversity of OxfordOxfordUK; ^6^ CEINGENaplesItaly; ^7^ Department of Molecular Medicine and Medical BiotechnologyUniversity of Naples Federico IINaplesItaly; ^8^ Keires AGBaselSwitzerland

## Abstract

Adenoviral vectors encoding hepatitis C virus (HCV) nonstructural (NS) proteins induce multispecific, high‐magnitude, durable CD4^+^ and CD8^+^ T‐cell responses in healthy volunteers. We assessed the capacity of these vaccines to induce functional HCV‐specific immune responses and determine T‐cell cross‐reactivity to endogenous virus in patients with chronic HCV infection. HCV genotype 1‐infected patients were vaccinated using heterologous adenoviral vectors (ChAd3‐NSmut and Ad6‐NSmut) encoding HCV NS proteins in a dose escalation, prime‐boost regimen, with and without concomitant pegylated interferon‐α/ribavirin therapy. Analysis of immune responses *ex vivo* used human leukocyte antigen class I pentamers, intracellular cytokine staining, and fine mapping in interferon‐γ enzyme‐linked immunospot assays. Cross‐reactivity of T cells with population and endogenous viral variants was determined following viral sequence analysis. Compared to healthy volunteers, the magnitude of HCV‐specific T‐cell responses following vaccination was markedly reduced. CD8^+^ HCV‐specific T‐cell responses were detected in 15/24 patients at the highest dose, whereas CD4^+^ T‐cell responses were rarely detectable. Analysis of the host circulating viral sequence showed that T‐cell responses were rarely elicited when there was sequence homology between vaccine immunogen and endogenous virus. In contrast, T cells were induced in the context of genetic mismatch between vaccine immunogen and endogenous virus; however, these commonly failed to recognize circulating epitope variants and had a distinct partially functional phenotype. Vaccination was well tolerated but had no significant effect on HCV viral load. *Conclusion:* Vaccination with potent HCV adenoviral vectored vaccines fails to restore T‐cell immunity except where there is genetic mismatch between vaccine immunogen and endogenous virus; this highlights the major challenge of overcoming T‐cell exhaustion in the context of persistent antigen exposure with implications for cancer and other persistent infections. (Hepatology 2016;63:1455‐1470)

**Abbreviations**
CMV: cytomegalovirus
DAA: direct‐acting antiviral
DMSO: dimethyl sulfoxide
ELISpot: enzyme‐linked immunospot
HCV: hepatitis C virus
HLA: human leukocyte antigen
IFN: interferon
IL: interleukin
nAb: neutralizing antibody
NS: nonstructural
PBMC: peripheral blood mononuclear cell
PCR: polymerase chain reaction
PEG: pegylated
RIB: ribavirin
SFC: spot‐forming cell
SI: stimulation index
TNF‐α: tumor necrosis factor‐α
vp: viral particle

Hepatitis C viral (HCV) infection is a global epidemic and a leading cause of death and morbidity from liver disease. Recent estimates show a seroprevalence of 2.8%, with 185 million people infected.^1^ While the incidence rate of HCV infection is decreasing in the developed world, HCV‐related deaths from advanced liver disease are predicted to increase over the next two decades.^2^

We recently developed an HCV T‐cell vaccine based on a chimpanzee adenovirus (ChAd3‐NSmut) and an adenovirus derived from a rare human serotype (Ad6‐NSmut), both encoding the nonstructural (NS) proteins of HCV genotype 1b, assessed in a heterologous prime/boost vaccination strategy in healthy volunteers.^3^, ^4^ The vaccine was safe and well tolerated, and the magnitude and breadth of T cells induced after a single priming vaccination were the most potent described in human HCV studies to date. We now assess the capacity of the same vaccine strategy to induce T cells in patients chronically infected with HCV genotype 1.

HCV may be particularly susceptible to a T‐cell vaccine, as evidenced by human leukocyte antigen (HLA) genetic association studies,^5^ chimpanzee T cell‐blocking experiments,^6^, ^7^ and the temporal association of the magnitude and breadth of the T‐cell response with viral eradication.^8^ In general, broad, high‐magnitude T‐cell responses are seen in primary HCV infection^9^ and are maintained in people who spontaneously resolve infection.^10^ However, once persistent disease is established, T‐cell responses are generally weak and narrowly focused^10^; and although they may be detected *ex vivo* and expanded *in vitro*,^11^ they are generally dysfunctional.^12^ To date, the capacity of an immunogenic T‐cell vaccine to recover T‐cell immunity through vaccination in the periphery, remote from the tolerogenic liver environment, in people with persistent HCV infection is unexplored.

Previous immunotherapeutic vaccine approaches have included HCV peptide vaccines,^13^ recombinant yeast expressing an HCV NS3‐core fusion protein,^14^ autologous dendritic cells loaded *ex vivo* with lipopeptides,^15^ and DNA vaccines encoding HCV proteins.^16^ In each case transient, very low‐level effects were seen on T‐cell induction or HCV viral load. More recently repeated vaccination in HCV‐infected patients with modified vaccinia Ankara encoding HCV NS proteins in combination with pegylated interferon‐α (PEG‐IFN‐α)/ribavirin (RIB) was associated with the induction of HCV‐specific T cells at low level with a nonsignificant increase in sustained virological response in the vaccinated group.^17^, ^18^ However, previous studies of HCV immunotherapy have not evaluated the effect of vaccination in the context of circulating viral variants.

In this study we determine the capacity of a T‐cell vaccine to induce HCV‐specific T cells in patients with chronic HCV infection. We assess vaccination in the setting of both high and low viral loads following treatment with PEG‐IFN‐α/RIB because mouse studies of lymphocytic choriomeningitis viral infection have suggested that T‐cell responses may be best recovered after viral suppression.^19^ We also assess in detail the relationship between T‐cell induction and endogenous circulating viral variants before vaccination. Our findings have important implications not only for HCV vaccine strategies but also for immunotherapy against other persistent pathogens and cancer.

## Patients and Methods

#### Patient Enrollment

Patients aged 18‐65 with HCV genotype 1 were eligible for inclusion. Patients with human immunodeficiency virus, hepatitis B virus, immunosuppressive illness, Ad6 or ChAd3 neutralizing antibody (nAb) titer >200, or evidence of cirrhosis (clinical, biochemical, or histological) were excluded (for patient demographics, see Supporting Table S1). Patients were recruited at Oxford University NHS Trust and Queen Elizabeth Hospital Birmingham, Birmingham, UK. The study (EudraCT: 2008‐006127‐32) was approved by the Medicines and Healthcare products Regulatory Agency, registered in the ClinicalTrial.gov database (ID: NCT01094873), and ethically approved (GTAC reference: GTAC162). All volunteers gave written informed consent, and the studies were conducted in accordance with good clinical practice.

#### Study Design

Study groups and vaccination regimes are detailed (Table 1). Forty volunteers were screened and 35 enrolled into arm A (n = 27), receiving concurrent PEG‐IFN‐α/RIB therapy (48 weeks), or arm B (n = 8), receiving vaccine alone. Patients in arms A1‐A3 received 5 × 10^8^, 5 × 10^9^, or 2.5 × 10^10^ viral particles (vp) ChAd3‐NSmut prime/Ad6‐NSmut boost 10 weeks apart, 14 weeks after starting PEG‐IFN‐α/RIB. All patients in A1‐A3 had a >2log drop in viral load before vaccination. Arm A4 received 2.5 × 10^10^ vp 2 weeks after starting PEG‐IFN‐α/RIB. Arm A5 received two priming vaccinations with ChAd3‐NSmut given 4 weeks apart before Ad6‐NSmut boost vaccination 10 weeks after the second priming vaccine, with the first vaccine given 14 weeks after the start of PEG‐IFN‐α/RIB. Arm A6 received the same vaccination schedule as arm A5 but with the first vaccination given 2 weeks after the start of PEG‐IFN‐α/RIB. Arm B groups received, respectively, 5 × 10^8^, 5 × 10^9^, or 2.5 × 10^10^ vp, with ChAd3‐NSmut prime/Ad6‐NSmut boost given 10 weeks apart without concomitant PEG‐IFN‐α/RIB. Vaccines were administered intramuscularly.

**Table 1:** Study Design HCV002

Arm	Patients (n)	IFN/Rib	ChAd3 Prime (Week)	ChAd3 Second Prime (Week)	Ad6 Boost (Week)	Dose of All Vaccines	Follow‐Up (Months)
A1	3^a^	+	14	—	24	5 × 10^8^ vp	18
A2	2^b^	+	14	—	24	5 × 10^9^ vp	18
A3	6	+	14	—	24	2.5 × 10^10^ vp	18
A4	6	+	2	—	12	2.5 × 10^10^ vp	18
A5	4	+	14	18	28	2.5 × 10^10^ vp	18
A6	4	+	2	6	16	2.5 × 10^10^ vp	18
Patients receiving vaccination alone (no IFN/RIB)							
B1	2	‐	4	—	14	5 × 10^8^ vp	9
B2	2	‐	4	—	14	5 × 10^9^ vp	9
B3	4	‐	4	—	14	2.5 × 10^10^ vp	9

a One volunteer received prime vaccine only and was replaced (021).

b One volunteer in this arm received prime only (031).

Two volunteers withdrew from arm A2 prior to vaccination after starting IFN‐α, and two patients received prime vaccine only (021 rash postprime, 031 difficulties in venesection). Overall, 23 patients in arm A received both vaccines. All volunteers in arm B received both vaccines. Five patients stopped PEG‐IFN‐α/RIB early: three with IFN‐α‐related side effects (16‐30 weeks), one after virological breakthrough (24 weeks), and one for personal reasons unrelated to the study (12 weeks). Volunteers documented all symptoms and recorded temperature daily. Solicited and unsolicited adverse events were recorded. All volunteers who received any vaccine were included in the analysis.

#### Adenoviral Constructs

The Ad6 and ChAd3 vectors encoding NS3‐5B (1985 amino acids) of genotype 1b BK strain HCV (accession number M58335) have been described^20^ and were manufactured at the Clinical BioManufacturing Facility, Oxford University, Oxford, UK.

#### Peptides and Antigens

We divided 494 peptides 15 amino acids long overlapping by 11 amino acids (BEI Resources) into six pools (F‐M) corresponding to NS3p, NS3h, NS4, NS5A, NS5B I, and NS5B II (mean 82, range 73‐112 peptides/pool) matching HCV genotype 1B strain BK. For cross‐reactivity assays peptides were synthesized by ProImmune (Oxford, UK). PepTivatorAdV5 Hexon pool was supplied by Miltenyi Biotec. Peptides were used at 3 μg/mL and 1 μg/mL for enzyme‐linked immunospot (ELISpot) and intracellular cytokine staining, respectively.

#### IFN‐γ‐ELISpot Assays

IFN‐γ‐ELISpot assays were performed *ex vivo* in triplicate at 2 × 10^5^ peripheral blood mononuclear cells (PBMCs)/well. PBMCs were separated by density gradient and counted using a Guava Personal Cell Analyser (Merck Millipore). Internal controls were dimethyl sulfoxide (DMSO) and R10 (negative controls) and concanavalin A, FEC (HLA class I‐restricted peptides from influenza, Epstein‐Barr virus, and cytomegalovirus [CMV]), and CMV lysate (Virusys Corporation; positive controls).

To determine a positive cutoff, T‐cell responses to DMSO were analyzed in 58 individuals with chronic HCV genotype 1 (Supporting Fig. S1). An HCV‐specific response was defined as an IFN‐γ‐ELISpot response ≥39 spot‐forming cells (SFCs)/10^6^ PBMCs/pool (mean ± 3 standard deviations) and three or more times the background DMSO level. Positive pools were summed and background‐subtracted. Epitope specificities were determined using frozen PBMCs rested overnight, by dividing positive peptide pools into minipools of approximately 10 peptides, then mapping to 15‐amino acid‐long peptides and optimal‐length peptides. CD8^+^ T‐cell depletion IFN‐γ‐ELISpots were performed using individual peptides and negative depletion beads (Dynal magnets). Positivity in response to vaccination was defined as a 30% increase in preexisting responses or the induction of T cells targeting new antigens.

#### Intracellular Cytokine Staining

PBMCs at 1 × 10^6^ cells/100 μL were stimulated with peptide pools (F + G + H = NS3/4, I + L + M = NS5A/B) or phorbol 12‐myristate 13‐acetate/ionomycin (50 and 500 ng/mL, respectively) or unstimulated (DMSO, 5 ng/mL). Brefeldin‐A was added (10 μg/mL) 2‐4 hours later, and cells were incubated overnight (37°C), stained with fixable‐near infrared live/dead dye (Life Technologies), fixed, and permeabilized (Foxp3 Fix/Perm kit; BD Biosciences) with CD3‐Pacific Orange (1:50), CD4‐Qdot605 (1:200), CD8‐peridinin chlorophyll protein Cy5.5 (1:200), IFN‐γ‐AlexaFluor 700 (1:50), interleukin‐2 (IL‐2)‐allophycocyanin (1:25), tumor necrosis factor‐α (TNF‐α)‐phycoerythrin‐Cy7 (1:25), and IL‐17‐phycoerythrin (1:100; Becton Dickson Sciences and Invitrogen) in perm buffer (30 minutes). Flow cytometry was performed on a BD LSRII machine and analyzed using FlowJo v.9.5.2 (Treestar).

#### Thymidine Incorporation Proliferation Assay

Thymidine incorporation assays were performed *ex vivo* on fresh PBMCs, 2 × 10^5^/well (in triplicate), with HCV proteins (1 μg/mL; Mikrogen GMBh) and [^3^H] thymidine incorporation. A stimulation index (SI) ≥3 was considered positive.

#### HLA Class I Pentamer Staining

PBMCs were stained with HLA*0201 and HLA*0101 phycoerythrin‐labeled pentamers (ProImmune; 20 minutes in phosphate‐buffered saline), stained with live/dead dye, fixed (1% formaldehyde), and then stained with CD3‐Pacific Orange and CD8‐Pacific Blue; stained with either CCR7‐PeCy7, CD45RA‐fluorescein isothiocyanate, CD127‐allophycocyanin, or CD38‐PerCP5.5 and HLA‐DR‐AlexaFluor 700; or permeabilized and stained with PD1‐PeCy7, perforin‐fluorescein isothiocyanate, CD161‐allophycocyanin, granzyme A‐PerCP Cy5.5, or granzyme B‐AlexaFluor 700.

#### HCV RNA Quantification

HCV RNA levels (lower limit of quantification 15 IU/mL) and subtypes were determined at the Virology Laboratory, John Radcliffe Hospital Oxford (COBAS AMPLICOR HCV test v2).

#### HCV Viral Sequencing

Autologous virus was sequenced at baseline and viral relapse. Thawed plasma (500 μL) was pelleted by centrifugation (23,600*g* at 4ûC for 60 minutes) and resuspended in 140 μL of plasma. Viral RNA was extracted (Qiagen). RNA was reverse‐transcribed, and first‐round polymerase chain reaction (PCR) was performed using the Superscript III One‐Step RT‐PCR System (Invitrogen) with specific primers and PCR cycling conditions (Supporting Table S2). Second‐round PCR used High Fidelity Taq DNA polymerase (Roche). PCR products were gel‐purified or PCR‐purified (Qiagen). Products were sequenced bidirectionally using second‐round internal primers and Prism Big Dye (Applied Biosystems) on an ABI 3100 automated sequencer. Cycling conditions were 96ûC for 1 minute, followed by 30 cycles of 96ûC for 15 seconds, 50ûC for 10 seconds, and 60ûC for 4 minutes. Sequences were analyzed and aligned using Sequencher (v. 4.10.1) and Se‐AI (v.2.0a11 http://tree.bio.ed.ac.uk/software/).

#### Sequence Variability at T‐Cell Epitopes at the Population Level

HCV sequences were downloaded from the Los Alamos database (http://hcv.lanl.gov/content/index). Single variants with a prevalence of >5% were identified at each immunodominant epitope. Incomplete sequences and nongenotype 1a or 1b sequences were excluded.

#### nAbs to Adenoviral Vectors

nAb assays were performed as described using recombinant adenoviruses expressing the reporter gene SEAP.^21^

#### IL‐28B Genotyping

DNA was tested for polymorphism rs8099917 using the ABI TaqMan allelic discrimination kit (Applied Biosystems) where G is the protective allele and T is the risk allele, as described.^22^

#### Statistical Analysis

Data were assumed to have a nongaussian distribution. Nonparametric tests were used throughout: paired tests within an individual (Wilcoxon matched pairs signed rank test) or unpaired tests between individuals (Mann‐Whitney). For correlations, Spearman's *r* test was used. Two‐tailed *P* values were used throughout. *P* < 0.05 was considered significant. Prism v. 5.0d for Mac was used for analysis.

## Results

#### Patient Characteristics and Virological Outcome

Overall, 24 patients were male, median age 43 (range 24‐65) years, mean baseline alanine aminotransferase 54 (range 16‐151) IU/mL, and median baseline HCV RNA 1.13 × 10^6^ (range 3868‐11.65 × 10^6^) IU/mL. Of patients treated with PEG‐IFN‐α/RIB, 14 achieved sustained virological response, 10 had nonsustained virological response, and one was lost to follow‐up. IL‐28B genotyping showed TT n = 22, GT n = 9, and GG n = 1 (Supporting Table S1).

#### Vaccination With Ad6‐NSmut and ChAd3‐NSmut Is Well Tolerated

The primary endpoints of the study were safety and immunogenicity. Predominantly mild local and systemic side effects were reported. Overall vaccination was well tolerated, with no vaccine‐related serious adverse events (Supporting Fig. S2.1). A transient increase in liver transaminases was observed in two patients, peaking at 103 IU/L and 339 IU/L, respectively (Supporting Fig. S2.2). A liver biopsy was performed in patient 038, which showed mild nonspecific inflammation only, in keeping with HCV infection.

#### Heterologous Ch3Ad‐NSmut Prime/Ad6‐NSmut Boost Induces HCV‐Specific T‐Cell Responses

**Figure 1:**
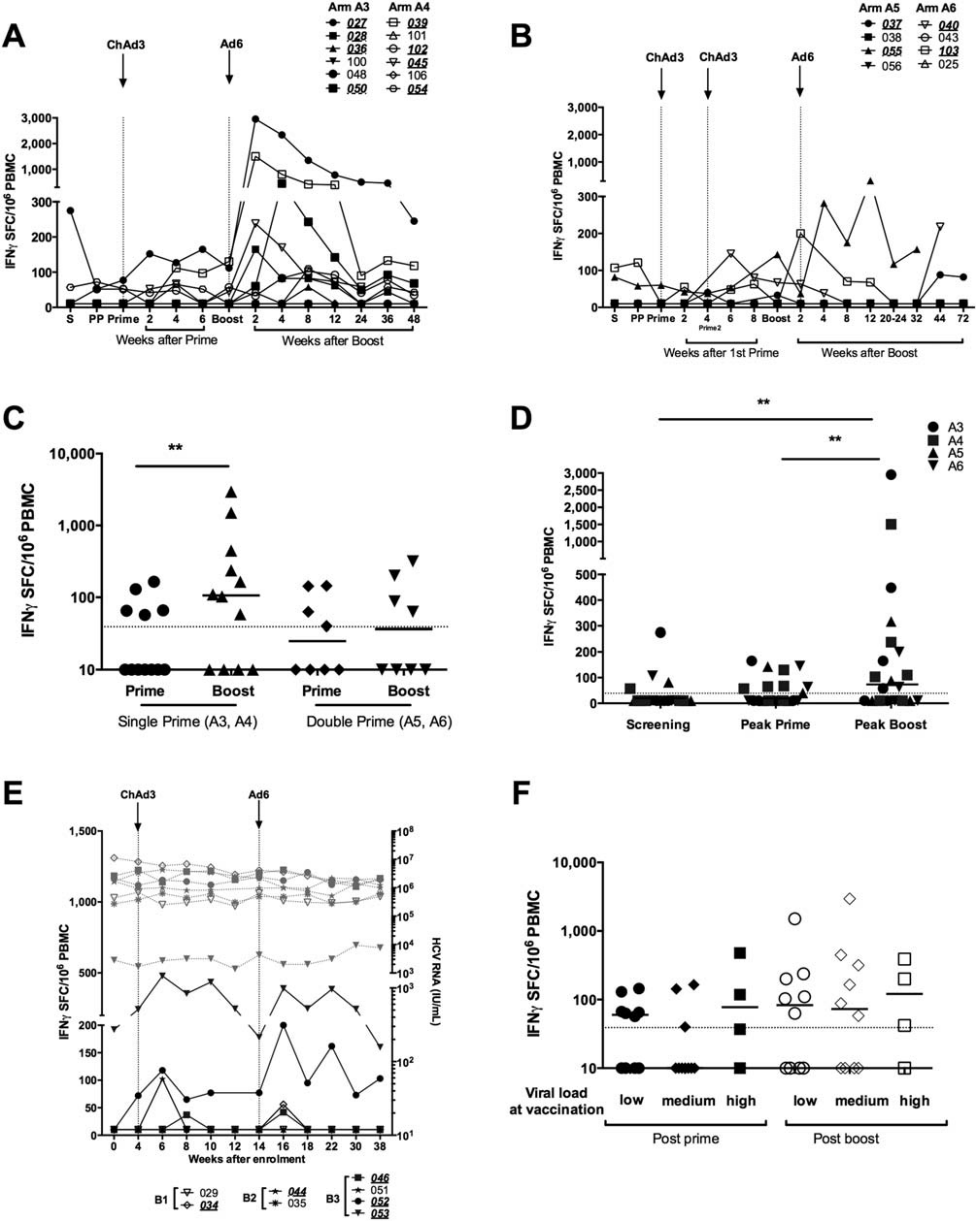
Magnitude of HCV‐specific T‐cell responses after ChAd3‐NSmut (prime) and Ad6‐NSmut (boost) vaccination. The total *ex vivo* IFN‐γ ELISpot responses across all NS peptide pools are shown. The kinetics of the response in individual patients receiving single‐prime (A) or double‐prime (B) vaccination (dose 2.5 × 10^10^ vp) with PEG‐IFN‐α/RIB. A comparative analysis of the magnitude of HCV‐specific T cells following single‐prime or double‐prime vaccination (C) and at baseline in comparison to peak postprime and peak postboost vaccination is shown (D). The kinetics of the HCV‐specific T‐cell response (solid lines) and HCV RNA (gray dotted lines) in individual patients receiving heterologous prime/boost vaccination at low, medium, and high doses (groups B1, B2, and B3, respectively; Table 1) without PEG‐IFN‐α/RIB (E). The magnitude of the T‐cell response in low (A4 and A6), medium (A3 and A5), and high (B3) viral load patient groups 14 or 2 weeks into or without PEG‐IFN‐α/RIB (all high‐dose vaccination; 2.5 × 10^10^ vp) (F). Dotted line at positive cutoff (C,D,F). Patients with a positive response are underlined (A,B,E). **P ≤ 0.01. Abbreviations: S, screening; PP, prepriming.

We first assessed the immunogenicity of ChAd3‐NSmut prime/Ad6‐NSmut boost vaccination in a dose‐escalation regime in patients receiving concurrent PEG‐IFN‐α/RIB (arm A). Vaccines were administered 10 weeks apart following a 2‐week or 14‐week lead‐in with PEG‐IFN‐α/RIB and as a single prime or double prime (ChAd3NS) with a heterologous boost (Ad6NS). No responses were seen at low and medium vaccine doses (data not shown). At the highest dose (2.5 × 10^10^ vp, arms A3 and A4; Table 1) HCV‐specific T cells were detected by IFN‐γ ELISpot in eight of 12 individuals receiving a single prime/boost (median 107 SFCs/10^6^ PBMCs, range 10‐2953), peaking 2‐4 weeks following boost vaccination (Fig. 1A). Double‐prime vaccination (4 weeks apart, arms A5 and A6) induced a response in four of eight vaccinees (median 36.5 SFCs/10^6^ PBMCs, range 10‐317) (Fig. 1B) and did not significantly enhance the response (Fig. 1C). Paradoxically, the highest responders were observed in the single‐prime arms. Overall, there was a significant increase in peak HCV T‐cell responses after boost but not after priming vaccination (Fig. 1D). A transient depletion of T cells specific for CMV, influenza, and Epstein‐Barr virus after PEG‐IFN‐α/RIB therapy has been described^23^; however, a clear effect of treatment on antiviral T‐cell responses was not observed in this study (Supporting Fig. S3).

Next, we assessed the effect of vaccination in untreated patients receiving prime/boost vaccination alone, 10 weeks apart (arm B) in a dose‐escalation strategy (5 × 10^8^, 5 × 10^9^, 2.5 × 10^10^ vp). At the low and medium doses two of four patients responded to vaccination. At the higher dose, T cells were induced in three of four patients (median postprime 78 SFCs/10^6^ PBMCs, range 10‐478, median postboost 121 SFCs/10^6^ PBMCs, range 10‐390). There was no effect of vaccination on HCV viral load (Fig. 1E).

#### Vaccination at High Versus Low HCV Viral Load

Mouse models of lymphocytic choriomeningitis virus have shown that therapeutic vaccination may be more efficacious after viral suppression.^13^ We compared vaccination 14 weeks after PEG‐IFN‐α/RIB therapy, when HCV RNA was undetectable in nine of 10 volunteers (arms A3, A5), with vaccination 2 weeks after therapy (arms A4, A6) when HCV viral loads were significantly higher (median 0 versus 11, 441 IU/mL, *P* = 0.0002) and in untreated patients in arm B, with high viral loads (median 2,300,000 IU/mL). There was no difference in vaccine responses between the three groups (Fig. 1F) and overall no correlation between the viral load at time of priming vaccination and the peak T‐cell response thereafter (Supporting Fig. S4).

#### HCV‐Specific T‐Cell Responses in Chronic HCV Infection Compared to Healthy Volunteers

**Figure 2:**
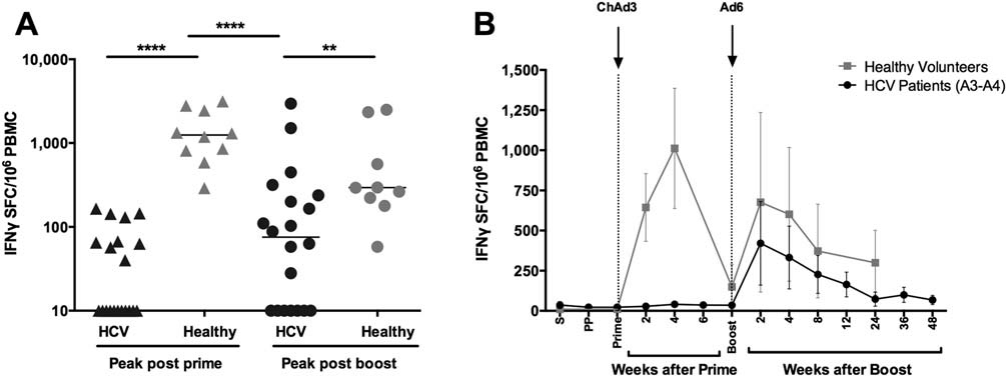
Magnitude of HCV‐specific T‐cell responses in healthy volunteers compared to HCV‐infected patients. A comparison of the magnitude of the T‐cell responses to HCV antigens after high‐dose vaccination with single/double ChAd3‐NSmut (prime) and Ad6‐NSmut (boost) (2.5 × 10^10^ vp) in individual healthy volunteers (gray n = 10; groups 7 and 10 as published^6^) and HCV patients (black, n = 20, groups A3‐6). Responses shown are the total positive *ex vivo* IFN‐γ ELISpot responses across all NS peptide pools at peak magnitude postprime and peak postboost. Bars at median (A). Kinetics of the total *ex vivo* IFN‐γ ELISpot response across all NS peptide pools in HCV‐infected patients (A3‐4) and healthy volunteers (group 10 as published^6^) receiving single‐prime and boost vaccination (B). Bars at mean and standard error of the mean. **P ≤ 0.01, ****P < 0.0001. Abbreviations: S, screening; PP, prepriming.

The magnitude of the response was significantly lower in HCV‐infected patients compared with healthy volunteers vaccinated with an identical regimen as part of a previous study^3^ (mean ± standard deviation HCV versus healthy volunteers: postprime 49.25 ± 54.15 versus 1476 ± 978.8, *P* < 0.0001, postboost 317.3 ± 704.7 versus 2183± 4633, *P* = 0.08, respectively) (Fig. 2A). The kinetics of the response also differed between these groups; the high‐magnitude response that was observed after priming vaccination in healthy volunteers was markedly attenuated in HCV‐infected patients (Fig. 2B).

#### Antivector Immunity in Response to Vaccination

**Figure 3:**
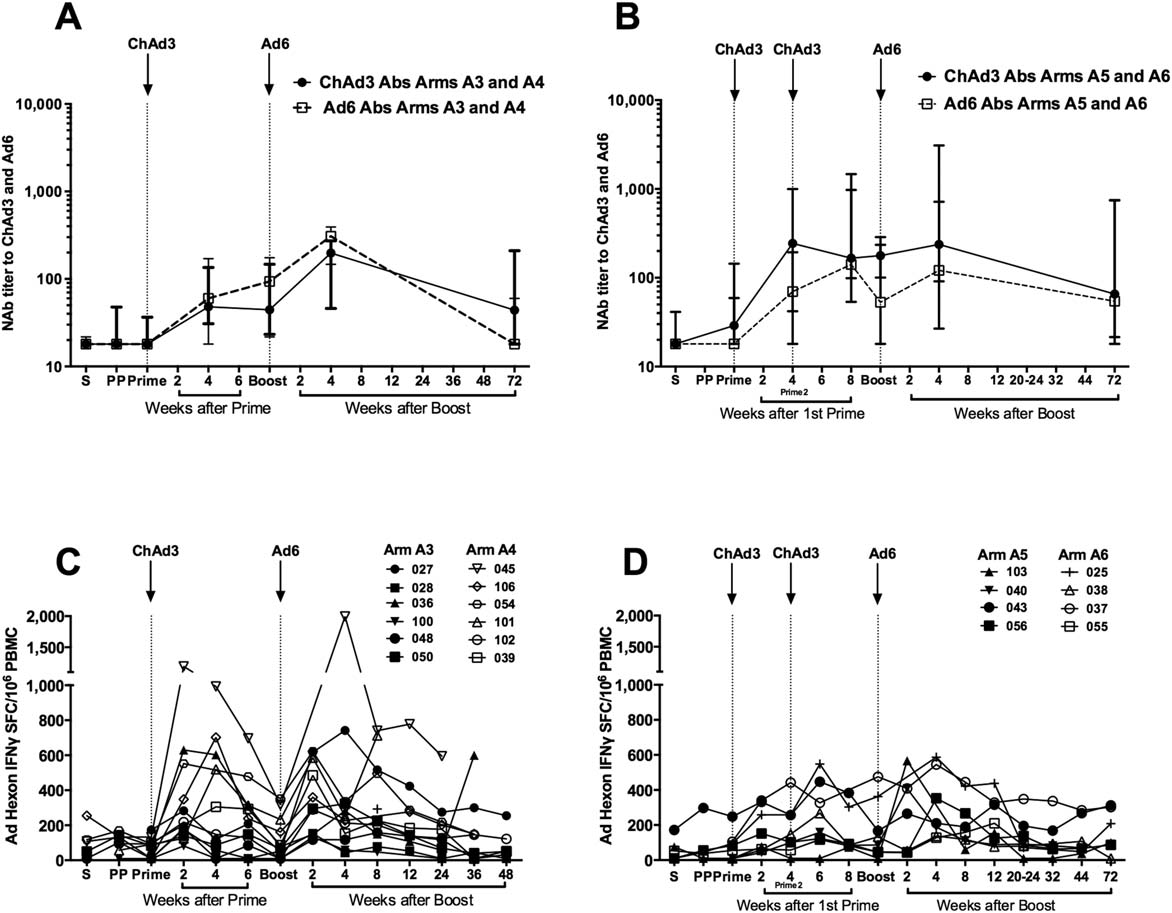
Antivector immunity after ChAd3‐NSmut (prime) and Ad6‐NSmut (boost) vaccination. Shown are nAbs to priming vector (ChAd3) and the heterologous boosting vector for single‐prime (A) and double‐prime (B) vaccinees as group data (dose 2.5 × 10^10^ vp), arms A3‐A6. The magnitude of antiadenovirus (Ad5 hexon proteins) T‐cell responses (by *ex vivo* IFN‐γ ELISpot) in patients receiving single‐prime (C) or double‐prime vaccination is shown (D). Abbreviations: S, screening; PP, prepriming.

Following priming vaccination, ChAd3 nAbs that were cross‐reactive with Ad6 were readily detectable and maintained to the time of heterologous boost in both single‐prime and double‐prime study groups (Fig. 3A,B). We found a negative correlation between anti‐ChAd3/Ad6 nAbs at the time of boost vaccination, with peak IFN‐γ‐ELISpot thereafter (*r* = ‐0.48, *P* = 0.03, and *r* = ‐0.44, *P* = 0.05) (Supporting Fig. S5). Robust antivector T‐cell responses were observed following both priming and heterologous boosting vaccination, showing that T‐cell responses are not generally defective in patients with HCV infection or during PEG‐IFN‐α/RIB therapy (Fig. 3C,D).

#### The Relationship Between Vaccine‐Induced T Cells and Endogenous Virus

T‐cell responses were mapped to individual peptides using IFN‐γ‐ELISpot. Fifteen antigenic targets were identified spanning the vaccine immunogen (Table 2; Supporting Fig. S6). Of these, 13 were CD8^+^ T‐cell epitopes that have been described in natural infection and two were novel CD8^+^ epitopes mapped to optimal length: NS5B_2804‐2814_ LTRDPTTPLAR and NS4B_1891‐1899_ ALVVGVVCA (patients 055 and 053, respectively) (Supporting Fig. S7). The most commonly identified epitope was NS3_1406_ (KLSGLGINAV), a well‐described HLA‐A2 epitope,^17^ targeted in six of 17 HLA‐A2^+^ patients. Depletion ELISpot and intracellular cytokine staining confirmed that mapped responses were CD8^+^. No mapped responses were made by CD4^+^ T cells.

**Table 2:** T‐Cell Responses Mapped to Individual Peptides/Pools

Group (Vaccines)	Patient	HLA‐A		HLA‐B		HLA‐C		Prevaccination‐Positive Pools (IFN‐γ SFCs/10^6^ PBMCs)	Postvaccination‐Positive Pools (IFN‐γ SFCs/10^6^ PBMCs, Week of Peak Response)	Mapped to Peptide (Parent Pool)
**A3 (weeks 14,24)**	**27**	30	2	44		7	5	F (40), G (45), H (170), L (120)	F (213, week 26), G (748, week 26), H (922, week 26), L (918, week 26), **M** (152, week 26)	CVNGVCWTV (F) KLSGLGINAV (G) LTTGSVVIVGRIILS/ SVVIVGRIILSGRPA (H) QEFDEMEECASHLPY (H) ILAGYGAGVAGALVA (H) RVCEKMALYDVVSTL (L) RPRWFMLCLLLLSVG (M)
	**28**	11	2	44	37	6	5	—	**G** (88, week 26) **H** (43, week 26), **M** (35, week 26)	KLSGLGINAV (G)
	**36**	3	2	44	51	16	14	—	**G** (59, week 32)	—
	**100**	24	2	13	60	6	10	—	—	—
	**48**	1	3	8	35	4	7	—	—	—
	**50**	2	30	8	49	7		—	**F** (50, week 38), **G** (388, week 28)	KLSGLGINAV (G)
**A4 (weeks 2, 12)**	**39**	24	30	13	7	6	7	—	**G** (80, week 14), **H** (85, week 14), **I** (794, week 14), **M** (510, week 14)	TPSPAPNYSRALWRV/ APNYSRALWRVAAEE (I) FQVGLNQYLVGSQLP (I) LSRARPRWFMLCLLL/ RPRWFMLCLLLLSVG (M)
	**101**	24	3	81	35	18	4	—	—	—
	**102**	68	3	27	39	1	7	M (52)	M (104, week 20)	—
	**45**	11	1	8	7	7		—	**G** (238, week 14)	HSKKKCDEL (G) VTLTHPITKYIMACM (G)
	**106**	3	29	35		4	8	—	—	—
	**54**	23	2	27	7	1	7	M (72)	M (110, week 20)	ARMILMTHF (M)
**A5 (weeks 14, 18, 28)**	**37**	1	2	49	44	5	7	—	**L** (40, week 18)	—
	**38**	66	2	41	18	7	17	—	—	—
	**55**	2	2	13	39	6	12	M (81)	**F** (88 week 36), **G** (112, week 36), M (143, week 36)	CVNGVCWTV (F) KLSGLGINAV (G) LTRDPTTPLAR (M)
	**56**	2	30	18	8	6	5	—	—	—
**A6 (weeks 2, 6, 16)**	**40**	24	2	60	7	7	10	—	**G** (145, week 8),	KLSGLGINAV (G)
	**43**	2	1	62	7	7	10	—	—	—
	**103**	2	2	44		5	16	F (120)	F (67, week 10), **G** (164, week 18)	CVNGVCWTV (F) KLSGLGINAV (G)
	**25**	2	68	18	65	7	8	—	—	—
**B3 (weeks 4, 14)**	**46**	1	2	8	44	4	7	—	**G** (42, week 16)	—
	**51**	11	32	18	51	2	5	—	—	—
	**52**	1	2	7	65	7	8	—	**G** (200, week 16)	GINAVAYYRGLDVSV (G)
	**53**	2	26	64		8		F (52), H (165), I (43)	F (92, week 8), **G** (50, week 6), H (270, week 6), I (81, week 6)	CVNGVCWTV (F) Fh (minipool only) ALVVGVVCA (G) Hf (minipool only)

Responses postvaccination are shown with “new” responses only detectable after vaccination highlighted in bold. The magnitude of the response and the study week of the response are given in parentheses. Responses mapped to the epitope or peptide level are shown.

**Figure 4:**
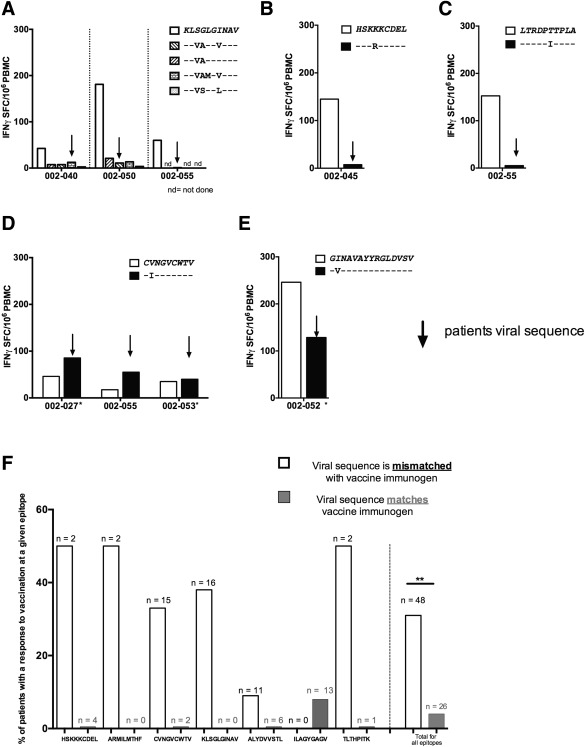
Cross‐reactivity of vaccine‐induced T cells to circulating viral antigen and the frequency of responders to vaccination when viral sequence is matched or mismatched with vaccine immunogen. T‐cell responses (IFN‐γ ELISpot) to peptide variants homologous to vaccine immunogen and circulating autologous virus at epitopes KLSGLGINAV (patients 040, 050, and 055) (A), HSKKKCDEL (patient 045) (B), LTRDPTTPLA (patient 055) (C), CVNGVCWTV (patients 027, 055, and 053) (D), and GINAVAYYRGLDVSV (patient 052) (E). Vaccine sequence in italics, with endogenous variants below. Arrows show patient's viral sequence. The percentage of HCV patients (with endogenous viral sequence that is completely homologous [matched] or mismatched with vaccine immunogen) who have a detectable T‐cell response (IFN‐γ ELISpot) at any time after vaccination to a given epitope target with known HLA restriction. Only patients with the corresponding HLA type are included. Data are presented for each epitope and as summary data (F). **P ≤ 0.01. Abbreviation: nd, not done.

Next we determined the endogenous viral sequence at the 15 CD8^+^ T‐cell epitopes at baseline and time of viral relapse in all patients (Supporting Table S3). At 14/15 epitopes, viral and vaccine immunogen sequences differed by one to four amino acids in patients targeting those epitopes (Table 3). We then assessed whether vaccine‐induced T cells could recognize endogenous viral peptide variants using IFN‐γ‐ELISpot and HLA class I pentamers. Vaccine‐induced T cells targeting the vaccine immunogen at NS3_1406_ KLSGLGINAV, NS3_1395_ HSKKKCDEL, and NS5B_2804_ LTRDPTTPLA failed to recognize circulating autologous variants at these epitopes (Fig. 4A‐C). In addition, T cells targeting the dominant NS3_1406_ epitope failed to recognize an additional two variant peptides (KLVALGINAV and KLVSLGLNAV) in the patient cohort. HLA class I pentamers (epitopes NS3_1406_ and NS3_1073_) matching either vaccine immunogen or endogenous variants confirmed the loss of cross‐reactivity to the NS3_1406_ KLSGLGINAV endogenous variants (Supporting Fig. S8). Partially cross‐reactive T‐cell responses between vaccine immunogen and endogenous viral sequences were observed at epitopes NS3_1073_ CVNGVCWTV and NS3_1411‐1425_ GINAVAYYRGLDVSV (Fig. 4D,E).

**Table 3:** HCV Viral Sequence at Mapped T‐Cell Epitopes in Patients Who Responded to Vaccination With Ch3Ad3‐NSmut/Ad6‐NSmut

Patient No.	Epitope/15‐mer Identified (HLA Restriction Where Known)	Viral Sequence at Epitope
**27**	CVNGVCWTV (A2) KLSGLGINAV (A2) LTTGSVVIVGRIILS/SVVIVGRIILSGRPA QEFDEMEECASHLPY ILAGYGAGVAGALVA RVCEKMALYDVVSTL (A2) RPRWFMLCLLLLSVG	C**I**NGVCWTV KL**VS**LG**L**NAV **C**VVIVGR**VV**L **R**EFDEMEEC**SQ**HLPY ILAGYGAGVAGALVA RVCEKMALYDVVS**K**L RPRWF**WF**CLLLL**AA**G
**28**	KLSGLGINAV (A2)	KL**VA**LGI/**V**NAV
**28B**	No detectable response	KL**VA**LG**V**NAV
**50**	KLSGLGINAV (A2)	KL**VA**LG**V**NAV
**50B**	No detectable response	
**39**	TPSPAPNYSRALWRV/APNYSRALWRVAAEE FQVGLNQYLVGSQLP LSRARPRWFMLCLLL/RPRWFMLCLLLLSVG	APNY**TF**ALWRV**SA**EEY F**R**VGL**HD**Y**P**VGSQLPnd
**45**	HSKKKCDEL (B8) VTLTHPITKYIMACM	HSK**R**KCDEL **II**LTHPITKYIMACM
**45B**	No detectable response	HSK**R**KCDEL **II**L/VTHPITKYIMACM
**54**	ARMILMTHF (B27)	**V**RMI**LL**THF
**54B**	No detectable response	nd
**055**	CVNGVCWTV (A2) KLSGLGINAV (A2) LTRDPTTPLAR	C**I**NGVCWTV KL**VA**LG**V**NAV LTRDP**I**TPLAR
**103**	KLSGLGINAV (A2)	KL**VA**LGINAV
**40**	KLSGLGINAV (A2)	KL**VAM**G**V**NAV
**40B**	No response to KLSGLGINAV CVNGVCWTV (A2)	KL**VAM**G**V**NAV C**I**NGVCWTV*
**53**	CVNGVCWTV (A2) ALVVGVVCA	C**I**NGVCWTVnd nd
**52**	GINAVAYYRGLDVSV	G**V**NAVAYYRGLDVSV

The amino acid sequence of the epitopes targeted in each patient is shown alongside the endogenous viral sequence in the patient. Nonhomologous amino acids are highlighted in bold with underlining.

*Denotes a T‐cell response seen after viral relapse, not vaccine‐induced.

Abbreviations: B, assessed after viral relapse; nd, not determined.

Sequence analysis at epitopes where HLA restriction was defined showed that genetic mismatch between immunogen and circulating virus was significantly associated with response to vaccination (*P* = 0.0063; Fig. 4F). For example, at the HLA‐B8 NS3_1395_ HSKKKCDEL epitope, circulating virus matched the immunogen in four of six HLA‐B8 patients; however, the only patient responding to this epitope (patient 045) had the well‐described escape variant NS3_1395_ HSK**R**KCDEL^24^ at baseline. Similarly, at epitope LTRDPTTPLAR, 25 viral sequences matched the immunogen but only the patient with a variant epitope (LTRDP**I**TPLAR) responded to vaccination (Supporting Table S3). However, genetic mismatch did not guarantee a response to vaccination, and no specific viral sequence at any epitope was predictive of a response to vaccination. For example, at epitope HLA‐A2 NS5_2594_ ALYDDVSTL identical endogenous epitope variants (ALYDVVS**K**L) were found in eight of 17 HLA‐A2 patients, only one of whom (027) had a T‐cell response to this epitope after vaccination. Similarly, at epitope HLA‐A2 NS3_1406_ KLSGLGINAV three of four variant epitopes identified in vaccine “responders” were also found in HLA‐A2 nonresponders (Supporting Table S3).

Using genotype 1a/1b sequences from the Los Alamos database, we evaluated the variability of the 15 vaccine‐induced T‐cell epitopes at the population level by Shannon entropy score (median 2149 sequences, range 1131‐3896/epitope). T‐cell epitopes were generally variable at the population level (Supporting Fig. S9A). The most dominant, NS3_1406_ KLSGLGINAV, was the most variable, with no single variant present in >30% of sequences at the population level (Supporting Fig. S9B). A minority of epitopes were found to be highly conserved (e.g., NS4B_1851_ ILAGYGAGV in 97.3% of genotype 1 sequences and homologous to vaccine immunogen; Shannon entropy score 0.35; Supporting Fig. S9C). However, at one conserved epitope, NS5B_2804_ LTRDPTTPLAR (Shannon entropy score 0.28), where 89% of population genotype 1 sequences were identical with the vaccine immunogen (Supporting Fig. S9D), the viral sequence in the only vaccinee responding to this epitope was a non‐cross‐reactive variant.

#### Vaccine‐Induced T‐Cell Function in Patients With Chronic HCV

**Figure 5:**
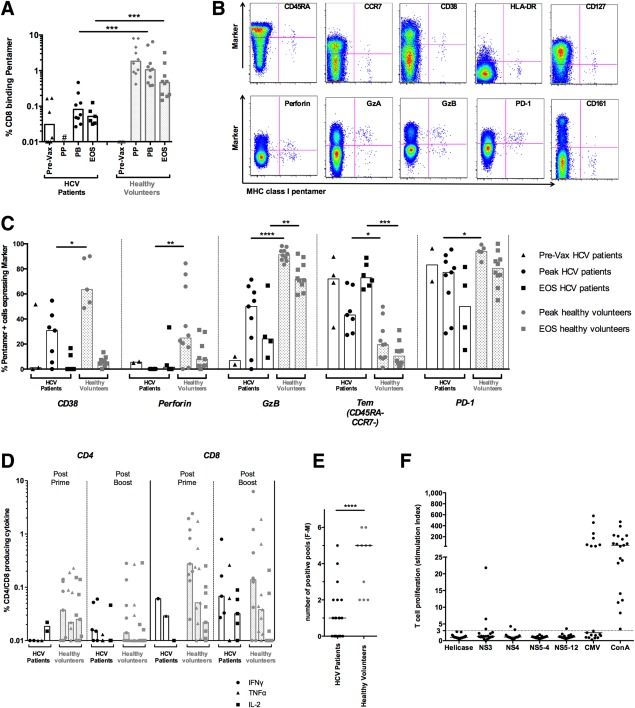
Comparison of phenotype and function of vaccine‐induced HCV‐specific T cells in patients versus healthy volunteers. Magnitude of HCV‐specific CD8^+^ T‐cell responses *ex vivo* detected by major histocompatibility complex class I pentamers (NS3_1436_ ATDALMTY, NS3_1406_ KLSGLGINAV, NS3_1073_ CVNGVCWTV) in healthy volunteers (gray) versus HCV‐infected patients (black) prevaccination, postprime 2‐4 weeks after ChAd3, 2‐8 weeks post‐Ad6 boost vaccination, and at the end of the vaccine study (EOS), 22‐50 weeks postboost. Bars at median. Mann‐Whitney *t* test (A). Representative fluorescence‐activated cell sorting plots are shown for major histocompatibility complex class I pentamer and marker staining (NS3_1406_ KLSGLGINAV) (B). Percentage of pentamer‐positive cells expressing a given marker in patients prevaccination (triangles), at the peak of the response (dots, 2‐4 weeks post‐Ad6 boost), or at the EOS (squares, 22‐50 weeks post‐Ad6 boost). For comparison the percentage of pentamer‐positive cells expressing a given marker in healthy volunteers (gray) receiving the same vaccinations is shown at the peak of the response (dots, 2‐4 weeks post‐ChAd3 prime) or at the EOS (squares, 22‐50 weeks post‐Ad6 boost). Bars at median. Mann‐Whitney *t* test (patient pre‐vax versus patient EOS, healthy peak versus patient peak, healthy EOS versus patient EOS) (C). Intracellular cytokine staining: PBMCs stimulated with peptide pools F + G + H (NS3‐4) or I + L + M (NS5). The percentage of total CD4^+^/CD8^+^ IFN‐γ, TNF‐α, or IL‐2 producing T cells at peak postboost vaccination (2‐8 weeks post‐Ad6) or postboost (2‐4 weeks post‐Ad6). Intracellular cytokine staining was performed on all patients with an IFN‐γ ELISpot response >150 SFCs/10^6^ PBMCs. All values are after background subtraction (DMSO wells), and only positive responses are shown. Bars at median. (D) Breadth of responses *ex vivo*: IFN‐γ ELISpot data for HCV‐infected patients versus healthy volunteers. Responses are the number of positive pools (see Materials and Methods) measured at peak magnitude during the study (E). Proliferative responses to HCV proteins: in patients 4 weeks post‐Ad6 boost, plotted as SI. SI ≥3 was defined as positive. Responses to positive control antigens (concanavalin A and CMV peptides) are shown. Bars represent the median (F). *P ≤ 0.05, **P ≤ 0.01, ***P ≤ 0.001, ****p < 0.0001. Abbreviations: EOS, end of the vaccine study; GzA, granzyme A; GzB, granzyme B; PB, post‐Ad6 boost; PP, postprime; pre‐vax, prevaccination.

We used HLA class I pentamers to examine the magnitude, phenotype, and function of vaccine‐induced T cells postboost and at the end of the study and compared these with published data using an identical vaccine regime in healthy volunteers^3^ (Fig. 5; Supporting Fig. S10). This analysis confirmed that the magnitude of the responses was lower in HCV‐infected patients than healthy volunteers at the epitope level (Fig. 5A; Supporting Fig. S10A). A subset of patients had small but detectable CD8^+^ T‐cell responses prevaccination, and changes in the phenotype of these T‐cell responses after vaccination were assessed and compared with healthy volunteers (Fig. 5C). The proportion of pentamer‐positive HCV‐specific T cells was high in patients for effector memory subset (CCR7^‐^/CD45RA) at baseline and remained significantly enriched after vaccination compared to healthy volunteers. There was no difference in bulk CD8^+^ effector memory subsets in patients compared to healthy volunteers (Supporting Fig. S10B). In patients, pentamer‐positive T cells were generally low in markers of cytotoxicity and activation (granzyme B, granzyme A, perforin, and CD38) at baseline; and although expression of these markers increased in response to vaccination, it remained significantly lower than that observed in healthy volunteers at peak response postboost. For granzyme B this difference was maintained to the end of the study. PD‐1 expression was high at baseline and remained high after vaccination in patients (Fig. 5C), although interpretation of this observation is complicated by the fact that PD‐1 expression is a marker of both exhaustion and activation. In healthy volunteers a small subset of vaccine‐induced T cells express CD161, a marker related to a more highly functional subset of antigen‐specific T‐cells.^25^ However, vaccine‐induced T cells in patients showed minimal CD161 expression. The expression of CD127 was also lower in patients compared to healthy controls at the end of the study (although not statistically significant) (Supporting Fig. S10C), suggesting a reduced capacity to persist by homeostatic proliferation in response to IL‐7. A strong positive correlation between the magnitude of response as measured by ELISpot and pentamer staining suggested that a population of pentamer‐positive cells that do not make IFN‐γ is not present in patients or volunteers (Supporting Fig. S10A).

Using intracellular cytokine staining, we showed that CD4^+^ T‐cell responses were detected at a low level only, while CD8^+^ T cells made some IFN‐γ and IL‐2 but minimal TNF‐α in patients (Fig. 5D; example plots in Supporting Fig. S10D).

Breadth of T‐cell response is an important correlate of immunological protection against HCV.^10^ In contrast to the broad responses detected in healthy volunteers, responses were significantly narrower in patients (median 5 positive pools, range 2‐6, versus 1 positive pool, range 0‐5; *P* < 0.0001 (Fig. 5E). Finally, although we detected robust proliferative responses to CMV control antigens in HCV‐infected patients (median SI 173.6, range 30.2‐1104.0), in stark contrast to data in healthy volunteers proliferative responses to HCV proteins were largely undetectable (median SI 3.5, range 3‐14.6) (Fig. 5F).

Overall these data suggest that T cells induced by vaccination in HCV‐infected patients remain at least partially functionally attenuated.

## Discussion

This is the first study to assess the capacity of a potent HCV T‐cell vaccine to restore antiviral T‐cell responses in patients with chronic HCV infection and to assess in detail the complex interplay between HCV‐specific T‐cell induction and circulating viral variants in the context of T‐cell immunotherapy. Although HCV‐specific T cells are generated in some patients, these do not typically target circulating virus because of an absence of sequence homology between circulating viral variants and vaccine immunogen at T‐cell epitopes. Furthermore, when T cells are induced, these appear to be functionally attenuated.

We have shown that an HCV T‐cell vaccine based on chimpanzee adenoviruses (ChAd3) and adenoviruses derived from a rare human serotype (Ad6) can induce high‐magnitude, polyfunctional CD4^+^ and CD8^+^ T cells targeting multiple HCV antigens after a single injection with either vaccine.^3^, ^4^ We found that when the same vaccines are given to patients with chronic disease, T‐cell responses were rarely detected after priming vaccination. Only after heterologous adenoviral boosting vaccination are T cells generated, but these are at a lower magnitude and target fewer antigens than we observed in healthy volunteers. Further analysis showed that while HCV‐specific CD8^+^ T cells could be generated in some people, HCV‐specific CD4^+^ T cells were largely absent.

In a stepwise approach, we vaccinated patients at low and intermediate HCV viral loads following PEG‐IFN‐α/RIB, before vaccinating treatment‐naive patients with high viral loads. Overall, vaccination was well tolerated with no clear evidence of liver immunopathology. This was expected as HCV infects only a minority of hepatocytes (<20%)^26^ and in the context of acute HCV infection, when T cells are maximally generated, severe hepatic inflammation is rare. While transient elevation in liver transaminases was observed in two patients, this was not clearly temporally related to vaccination and neither patient had a vaccine‐induced T‐cell response. Although murine models of lymphocytic choriomeningitis viral infection and other human models of persistent viruses support the hypothesis that T cells may be maximally induced in the context of viral suppression,^19^, ^27^ we found no correlation between HCV viral load and T‐cell induction. One confounder to consider is that both PEG‐IFN‐α and RIB, which were coadministered with vaccination, have been shown to attenuate cellular immune responses.^28^, ^29^ However, antivector, influenza, Epstein‐Barr virus, and CMV‐specific T‐cell responses could be detected in the presence of PEG‐IFN‐α/RIB therapy, suggesting that therapy did not induce a global impairment of T‐cell function.

It is well established that in chronic HCV disease, T cells are found at a low magnitude and are functionally attenuated with reduced proliferative capacity.^10^ The reasons for this may include persistent high‐level antigen exposure, inhibition by regulatory T cells, and dysfunctional T‐cell priming in the tolerogenic liver environment (reviewed by Bowen and Walker^30^). These defects appear to persist following viral eradication with PEG‐IFN‐α/RIB.^31^ We assessed whether a potent T‐cell vaccine could overcome these factors, either by enhancing existing weak responses or by generating new HCV‐specific T‐cell responses. However, we found that T‐cell responses were generated in a minority of individuals.

In order to better understand why some patients responded to vaccination and others did not, we evaluated the sequence of endogenous virus in patients. We showed that in vaccine responders there was sequence divergence between circulating viral sequence and vaccine immunogen at T‐cell targets, with a loss of cross‐reactivity between these variants. This suggests that HCV T‐cell vaccines may induce T‐cell responses in chronic infection but primarily in the absence of homologous antigen stimulation. The corollary of this is that these T cells will be unable to target endogenous virus or inhibit viral replication. The finding that vaccination had no effect on HCV viral load is in keeping with this. In contrast, T cells were generally not induced when there was sequence homology between the vaccine immunogen and endogenous virus at known T‐cell epitopes; in this context vaccination was unable to restore functional immunity. These observations are relevant for vaccine development against other variable pathogens such as human immunodeficiency virus and for cancer immunotherapy, where mismatch between vaccine immunogen and endogenous antigens will commonly arise.

Failure to respond to vaccine in the context of an epitope which is matched between virus and vaccine may be a result of T‐cell exhaustion. Detection of T‐cell responses where mismatch occurred may therefore either be the result of *de novo* priming or represent expanded memory responses, the latter potentially demonstrating the phenomenon of “original antigenic sin” (enhanced memory responses to cross‐reactive epitopes).^32^ Modifying the antigen to broaden the cross‐reactivity may recruit further T‐cell pools,^33^ but it will be challenging to overcome the limitations for therapeutic vaccination posed by T‐cell exhaustion and escape.

We also observed that T cells generated by vaccination were functionally impaired in HCV‐infected patients compared to healthy volunteers: low in proliferative capacity, markers of both cytotoxicity and T‐cell activation, and TNF‐α production, a phenotype that is characteristic of chronic viral exposure.^12^

The marked lack of T‐cell proliferation may reflect the fact that the proliferation assay uses recombinant HCV proteins that preferentially detect CD4^+^ T‐cell responses. However, it has also been shown that there is a hierarchical loss of function during T‐cell exhaustion, such that proliferative capacity may be lost relatively early after prolonged antigen exposure in comparison to IFN‐γ production.^34^ A further explanation for partial dysfunction of vaccine‐induced T cells is that HCV infection *per se* inhibits the priming of naive T cells to produce functional memory T cells. In support of this, it has been shown that HCV infection is associated with a loss of other virus‐specific (CMV) mature effector memory CD8^+^ T cells.^35^ However, in the absence of liver cirrhosis, there is little evidence that HCV‐infected patients respond abnormally to routine clinical vaccines^36^; and in our study patients generated robust antivector T‐cell responses following vaccination. An alternative explanation is that vaccination is not priming truly naive T cells but is stimulating memory responses that were generated early in infection but that were rendered partially dysfunctional following viral exposure. The degree of dysfunction would then depend on the duration of antigen exposure at the epitope level, with more functional responses associated with viral escape early in infection.

In this study vaccination induced predominantly CD8^+^ T cells. The generation of robust CD4^+^ T‐cell responses is thought to be a key determinant of viral clearance as evidenced both experimentally in natural history studies^37^ and through large host genome‐wide association studies that identify HLA class II alleles as critical genes associated with spontaneous resolution.^38^ Therefore, vaccine strategies that aim to induce robust CD4^+^ T‐cell immunity should be considered. Heterologous boosting with a modified vaccinia Ankara vector following adenoviral vector prime has recently been shown to induce a more balanced CD4^+^ and CD8^+^ response compared to heterologous adenoviral vaccination.^4^ This vaccine regimen is now in efficacy testing in intravenous drug user populations as a prophylactic HCV vaccine^39^ (NCT01436357) and is also being currently evaluated in an HCV therapeutic vaccine study (NCT01296451). The use of an alternative boosting vector also overcomes the issue of antiadenoviral antibodies that may limit T‐cell responses to a heterologous adenoviral boost.

The utility of an immunotherapeutic approach for HCV can be debated. Historically, treatment of HCV has relied on PEG‐IFN‐α/RIB‐based therapies associated with severe side effects. Recently licensed all‐oral combination direct‐acting antiviral (DAA) therapies given for 8‐24 weeks will transform our capacity to treat patients, with recent phase 2b/3 studies showing viral clearance rates of >90%.^40^ However, impacting on the HCV epidemic may require the treatment of intravenous drug users, who frequently live in chaotic social settings^41^; therefore, vaccines administered as adjuvant immunotherapy that markedly shorten therapy duration would be beneficial. In addition, the development of a prophylactic HCV vaccine remains an important goal, and the assessment of T‐cell induction in the presence of circulating viral variants may be readily assessed through therapeutic vaccine strategies and thus inform rational vaccine design. With the advent of all‐oral DAA therapies, the capacity of a T‐cell vaccine to induce a functional T‐cell response and thus protect individuals from reinfection after drug‐induced viral clearance will depend of the recovery of T‐cell function after therapy. Recent data suggest that both natural killer function and T‐cell function may recover after all‐oral DAA therapy, in contrast to IFN‐based treatments.^42^, ^43^ Therefore T‐cell vaccine studies in this group will be of particular interest.

Our study highlights the formidable challenges in developing an effective immunotherapeutic vaccine for HCV with implications for immunotherapy against variable pathogens in general and in cancer. Additional strategies, such as the use of checkpoint modulation, may be a useful adjunct,^44^ although the safety profiles of these are unlikely to be clinically acceptable for the treatment of HCV, where DAAs represent an alternative curative treatment strategy. In conclusion, we have shown that a highly potent T‐cell vaccine regimen is not able to restore T‐cell function during chronic HCV disease and that without determining circulating viral sequence for variable pathogens, the true value of T‐cell induction cannot be accurately assessed.

## Supporting information

Additional Supporting Information may be found at onlinelibrary.wiley.com/doi/10.1002/hep.28294/suppinfo.

Supporting Information
