# Supplementary material for: Chronic hepatitis C viral infection subverts vaccine‐induced T‐cell immunity in humans
Source: Hepatology. 2016 Jan 22;63(5):1455–70. doi: 10.1002/hep.28294 (PMC4842008; doi:10.1002/hep.28294)
Supplement: Supplementary file 1 — Supporting Information [file HEP-63-1455-s001.pdf]

Supplementary Figure S1: Determining the positive cut off for IFNγ-ELISpot in HCV patients

To determine a positive cut off for an HCV specific response, T-cell responses to DMSO were analysed in 58 individuals with chronic HCV genotype 1 infection (not on treatment) in IFNγ-ELISpot assays. An HCV specific response was then defined as a response ≥ mean + 3 x standard deviations (s.d) (≥ 39 SFC/10^6 PBMC /pool).

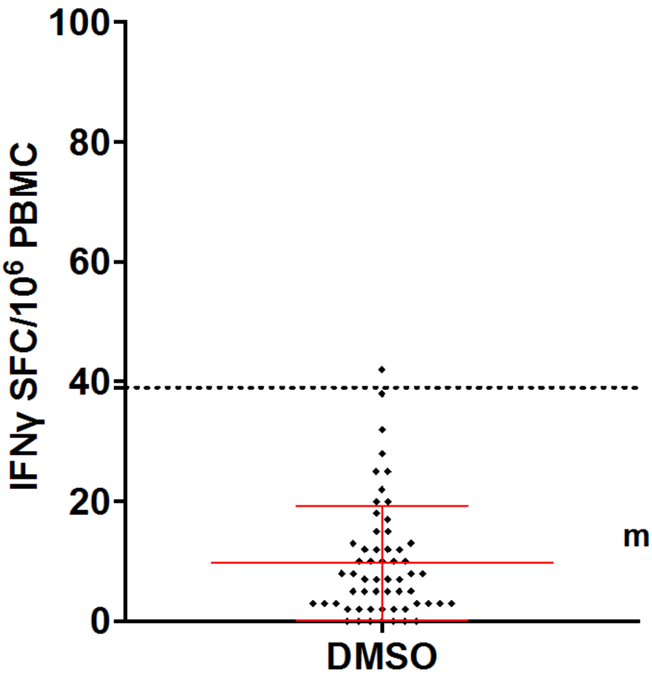

	DMSO
Number of values	58
Minimum	0.0
25% Percentile	3.000
Median	7.000
75% Percentile	13.00
Maximum	42.00
1% Percentile	0.0
99% Percentile	42.00
Mean	9.724
Std. Deviation	9.580

mean +3 x Std. Deviation = 39

Supplementary Figure S2.1: local and systemic adverse events

Safety data is shown following vaccination with ChAd3-NSmut (**A-D**) and Ad6-NSmut (**E-H**) in patients receiving concurrent PEG-IFNα/rib therapy (Arm A) and patients receiving vaccination alone (Arm B). The percentage of patients with local and systemic adverse events classified by type (**A, B, E, F**) and severity (**C, D, G, H**) is shown, and the number of patients receiving each vaccination indicated in parenthesis.

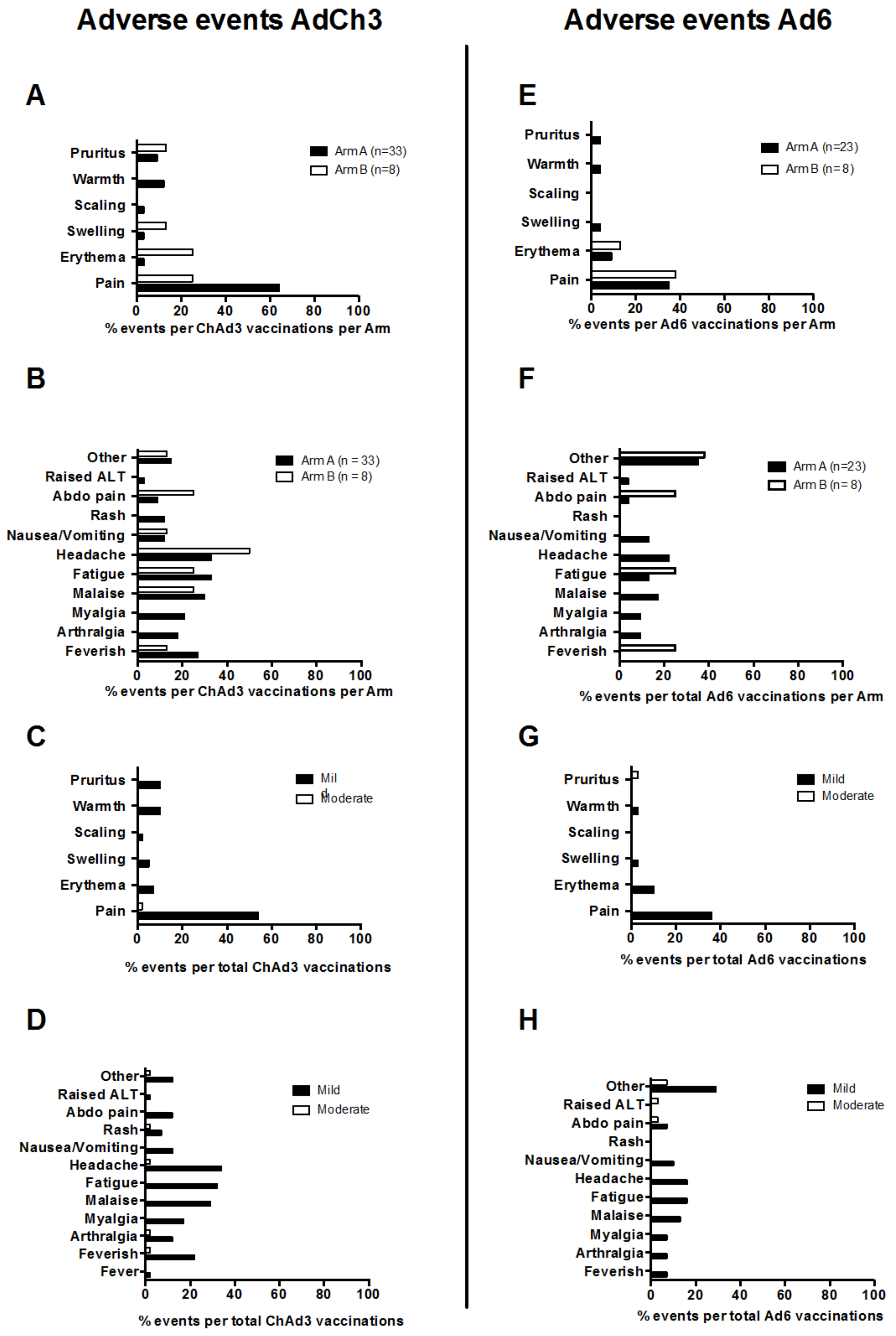

Supplementary Figure S2.2: Liver enzymes in relation to vaccination

A) Kinetics of Alanine transaminase (ALT) in relation to vaccination is shown in two patients with transient elevations in ALT. Patient 024 (Arm A2) received medium dose vaccination (5 x 10^9 vp) and 038 (Arm A5) received high dose vaccination (2.5 x 10^{10} vp). Liver biopsy of 038 showed non-specific inflammation.

B) Correlation between peak IFNγ ELISpot response at any time point and maximum change in ALT after vaccination (Peak ALT after vaccination – ALT at time of vaccination).

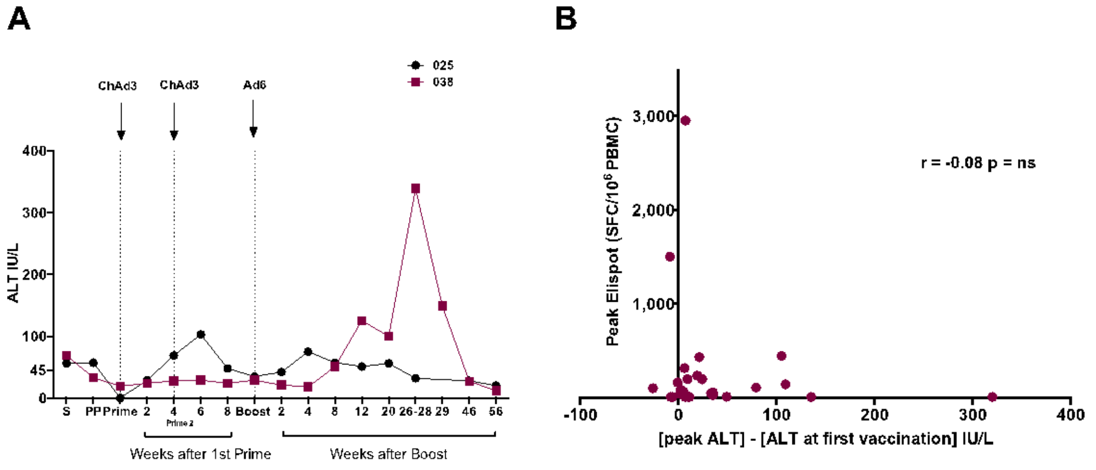

Supplementary Figure S3: The magnitude of non-HCV viral specific T-cells during the study

Responses shown are the *ex vivo* IFNγ ELISpot response to CMV lysate (**A**) or to a pool of influenza, EBV, and CMV (FEC) CD8+ T-cell epitopes (**B**; see Materials and Methods) in patients that showed a positive response to these peptides at any point during the trial (CMV n=8; FEC n=11). Bars represent the mean +/- SEM for patients in arms A3 - A6 receiving concurrent PEG-IFNα/rib therapy (period of therapy shaded in grey).

A

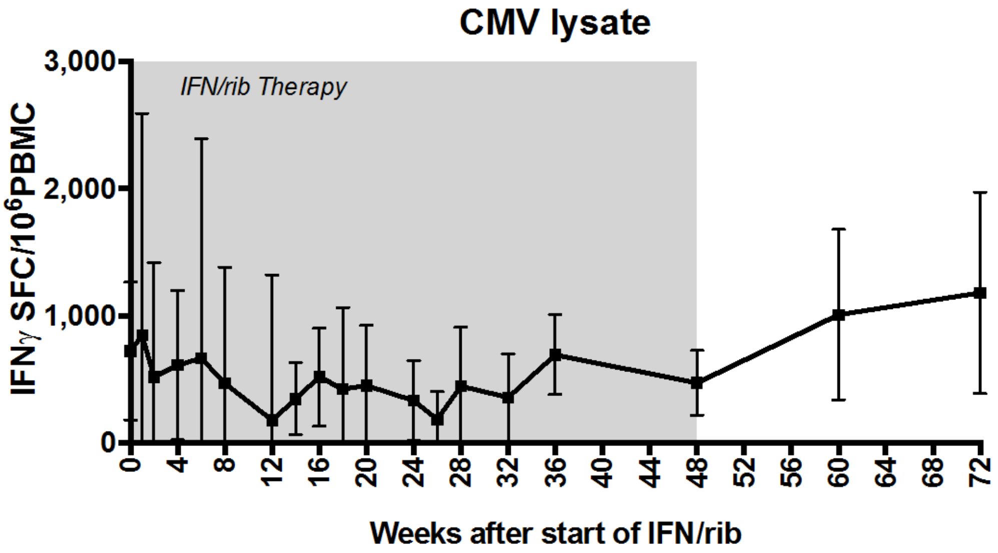

B

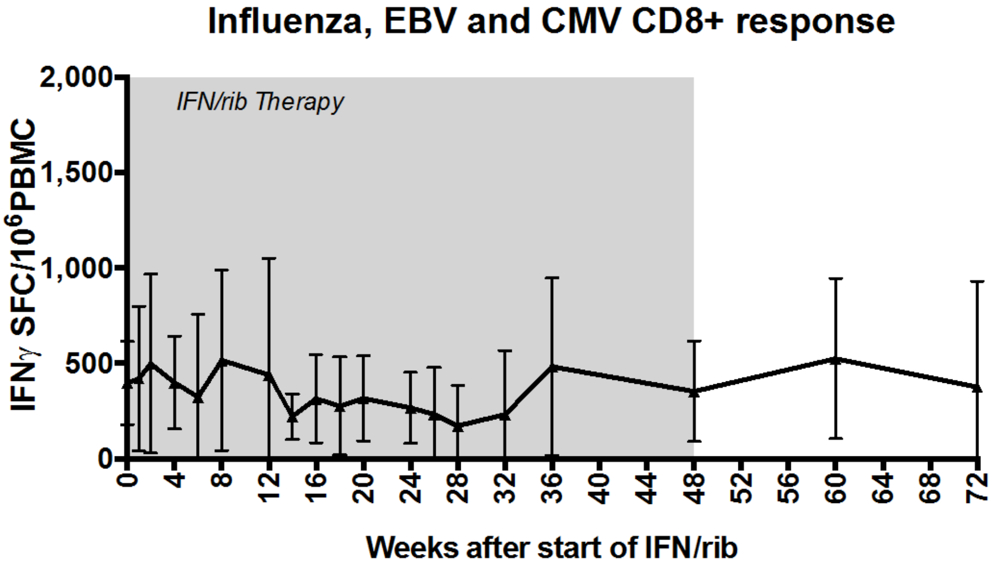

Supplementary Figure S4: Peak magnitude of response to vaccination does not correlate with viral load.

A correlation of HCV RNA (IU/mL) at time of prime vaccination with the peak HCV specific T-cell response for groups A3-6 is shown.

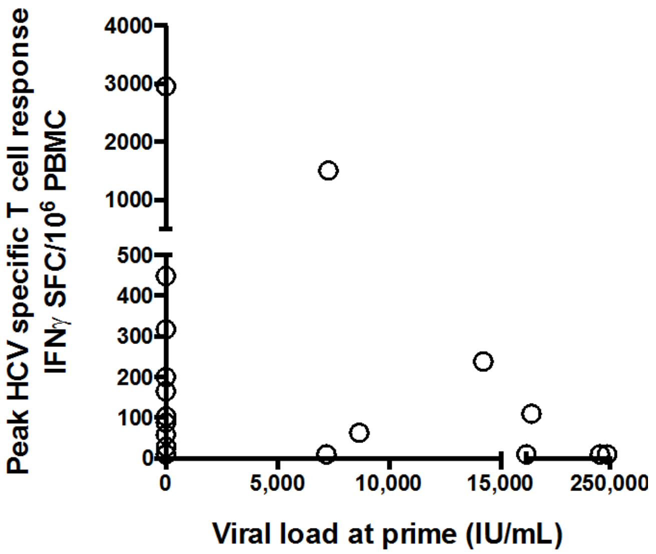

Supplementary Figure S5: Anti-vector immunity: Neutralising antibodies to hexon of Adenovirus. A correlation between the nAb titre to ChAd3 (**A**) and Ad6 (**B**) with the peak magnitude of the HCV specific T-cell response (*ex vivo* IFNγ ELISpot response across all NS peptide pools) 2-4 weeks after boosting vaccination is shown.

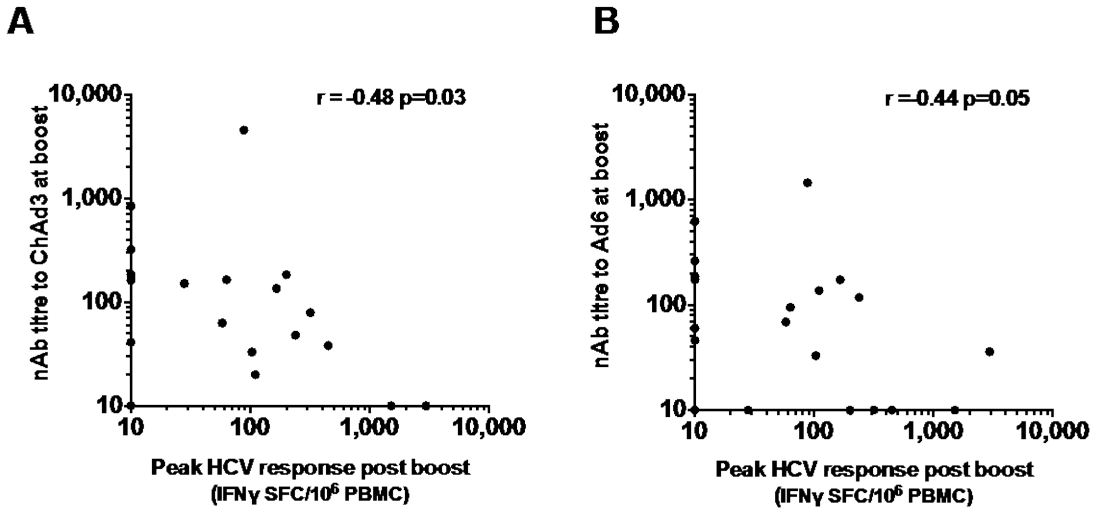

Supplementary Figure S6: Epitopes mapped to HCV genomic regions:

Epitopes targeted by vaccine induced T-cells are represented by different coloured vertical lines at the corresponding position along the HCV genome and the start position and amino acid sequence at each epitope indicated below.

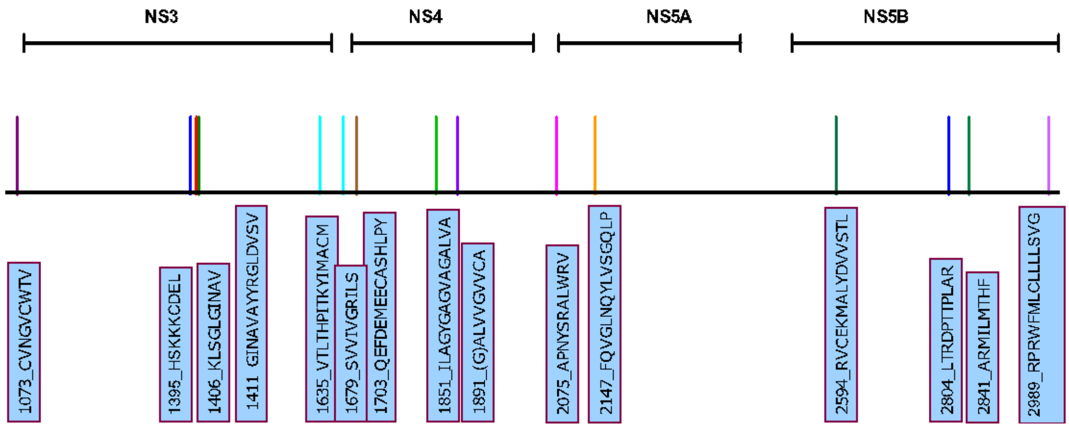

Supplementary Figure S7: Defining novel optimal epitopes

Optimal epitopes were defined to two new CD8+ T-cell peptides in IFNγ ELISpot assays using variant peptides at 3µg/ml followed by serial dilutions in patient 053 (Arm B3); peptide LSPGALVVGVVCAAI (NS5B$_{1887-1901}$) (**A** and **B)** and in patient 055 (Arm A5); peptide LTRDPTTPLARAAWE (NS5B$_{2804-2818}$) (**C** and **D**). TW= treatment week.

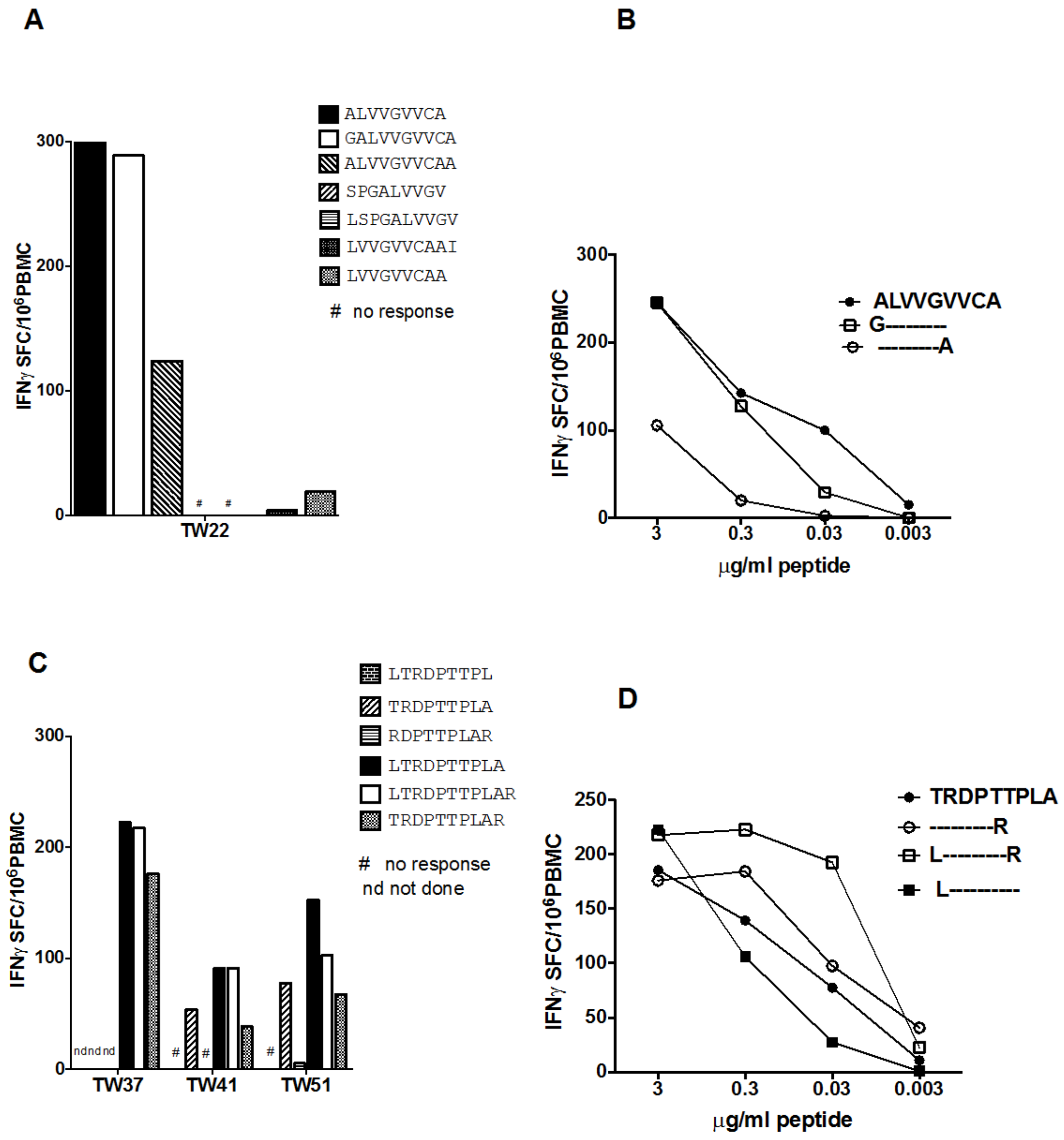

Supplementary Figure S8:

FACS analysis using HLA-A2 pentamers to assess cross-reactivity of T-cells induced by the vaccine immunogen at epitopes KLSGLGLINAV ($NS3_{1406}$; left panels) and CVNGVCWTV ($NS3_{1073}$; right panels). Analysis using peptides corresponding to the vaccine immunogen, and endogenous viral sequence is given. % pentamer+ $CD8^{+}$ T-cells is represented in the top right of each plot. Cells are gated on live $CD3^{+}$ cells.

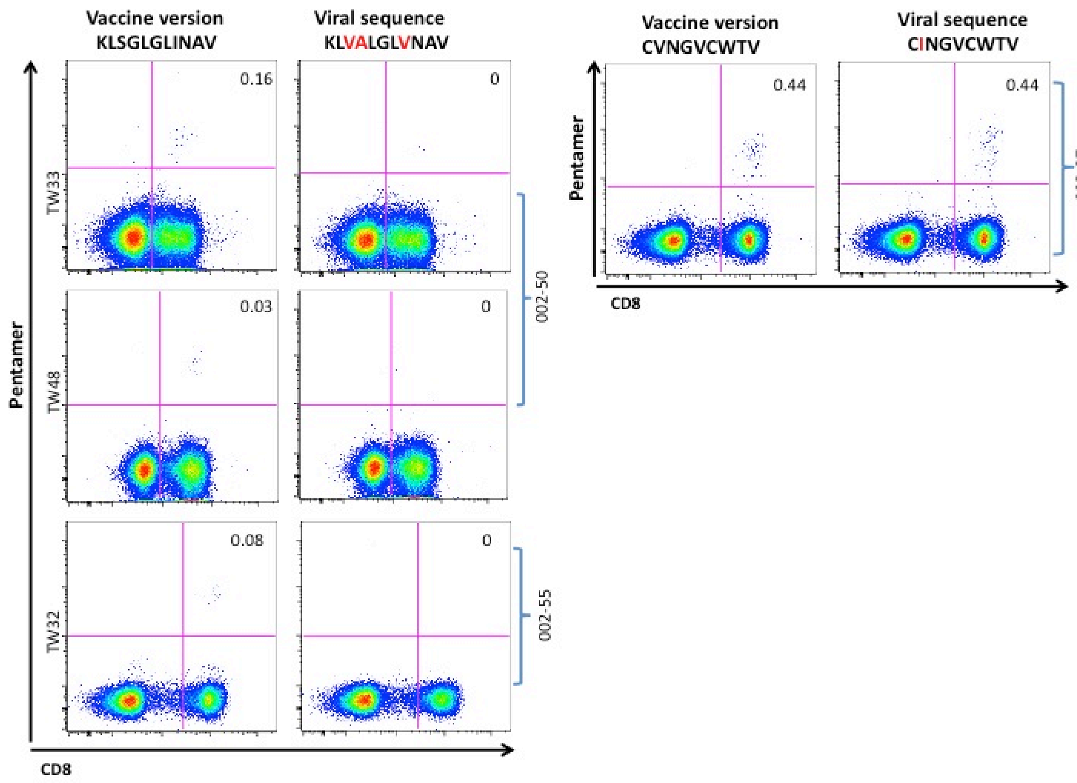

Supplementary Figure S9: Viral variability at a population level of HCV T-cell epitopes

The degree of variability in genotype 1 HCV sequences at identified epitopes in patients vaccinated with ChAd3-NSmut (prime)/Ad6-NSmut (boost) is defined using Shannon Entropy in a median of 2149 sequences (range 1131-3896) for each epitope derived from the Los Alamos database. A low Shannon entropy score indicates a conserved epitope (**A**). The population frequency of HCV sequence variants at 4 epitopes (**B-E**) grouped by subtype 1a or 1b is given. The variant that corresponds to the vaccine immunogen version is underlined (x axis).

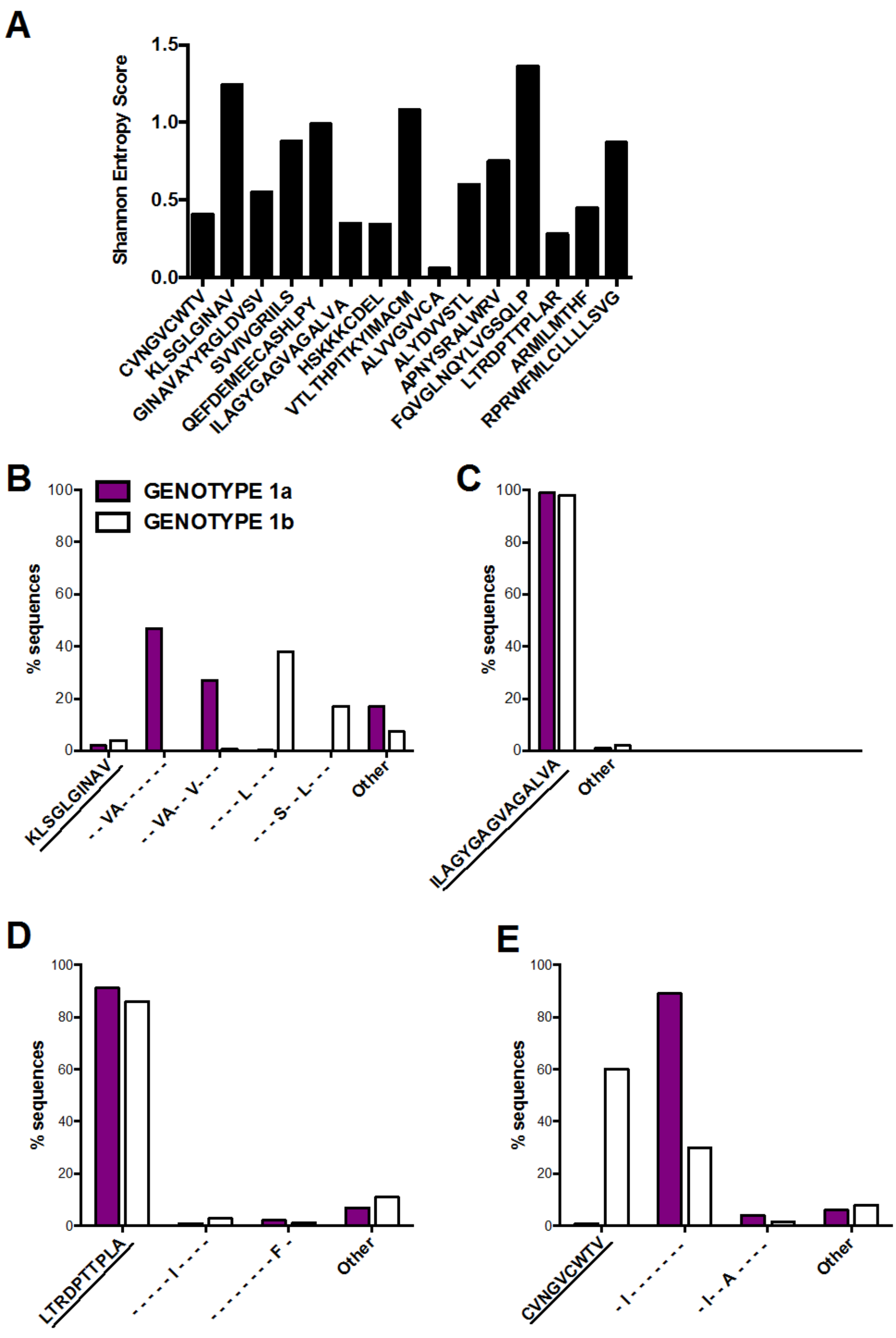

Supplementary Figure S10: Magnitude and Phenotype of HCV-specific T-cells. (**A**) The magnitude of the HCV-specific T-cell response to vaccination correlating ELISpot vs. pentamer staining. Spearmans rank: healthy volunteers r = 0.8207 P<0.0001; patients r = 0.7443 p=0.0108. (**B**) A comparison of the bulk CD8+ T-cell memory subsets in healthy volunteers vs. patients: Naïve-like (CD45RA+CCR7+), central memory (Tcm: CD45RA-CCR7+), terminal effector memory (Temra: CD45RA+CCR7-), and effector memory (Tem: CD45RA-CCR7-). (**C**) % of pentamer+ cells expressing a given marker in patients pre-vaccination (triangles), at the peak of the response (dots, 2-4weeks post Ad6 boost) or at the end of the study (EOS; squares, 22-50 weeks post Ad6 boost). For comparison the % of pentamer+ cells expressing a given marker in healthy volunteers (grey) receiving the same vaccinations are shown at the peak of the response (dots, 2-4weeks post ChAd3 prime) or at the EOS (squares, 22-50 weeks post Ad6 boost). Bars at median. Mann-whitney t-test (patient pre-vax vs patient peak, patient pre-vax vs patient EOS, healthy peak vs Patient peak, healthy EOS vs Patient EOS). Pre-vax = pre-vaccination. GzA = granzyme A. (**D**) Example FACS plots showing staining of TNFα/IFNγ for CD4+ and CD8+ T-cells after culture with DMSO or stimulation with NS3-4 or NS5. Patient number and treatment week are shown.

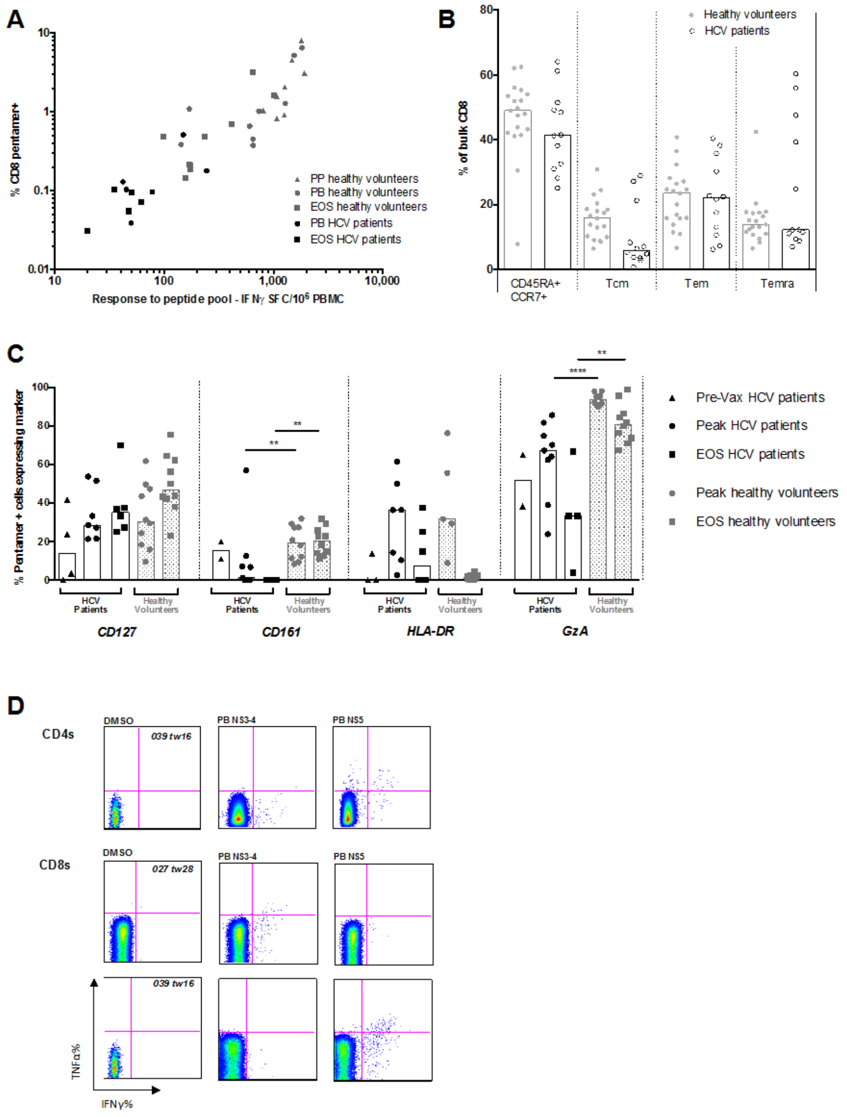

Supplementary Table S1: Patient demographics and treatment outcome

Duration of IFN/RBV therapy and outcome of therapy in all vaccinated patients is shown .Fibrosis scores were determined by pre-treatment liver biopsies or by fibroscan*. Undetectable viral load by PCR after 4 weeks of therapy (RVR), 12 weeks of therapy (EVR) and 6 months following end of therapy (SVR) are shown. IL28B status was determined at SNP rs80990917. (NA= not applicable, nd=Not done, FU=follow up). ALT at baseline IU/L.

Patient	Group	Sex	Age	HCV subtype	Fibrosis Score: Ishak or *Kpa	ALT	Treatment (IFN/RBV) Duration (weeks)	Baseline HCV RNA (IU/ml)	RVR	EVR	SVR	IL28B (SNP rs8099917)
21 (prime only)	A1	F	65	1a	3	28	48	$1.98x10^6$	Y	Y	Y	nd
20	A1	M	54	1	3	40	48	$1.54x10^6$	N	Y	Y	TT
30	A1	M	56	1a	4	28	48	15995	Y	Y	Y	TT
31 (prime only)	A2	M	36	1a	2	56	48	$6.3 x10^6$	N	N	N	TT
24	A2	M	42	1b	3	69	48	$11.3x10^6$	Y	Y	Y	TT
28	A3	M	58	1a	2	144	48	$4.14x10^6$	N	N	N	TT
48	A3	M	53	1a	4	84	48	$2.93x10^6$	N	N	N	TT
50	A3	M	50	1a	1	42	48	761224	N	N	N	GT
100	A3	M	24	1b	7.5*	54	48	$1.31x10^6$	N	Y	Lost to FU	GG
27	A3	F	33	1	2	18	48	22228	Y	Y	Y	GT
36	A3	F	32	1b	nd	16	30	9199	Y	Y	Y	TT
101	A4	M	43	1a	6*	71	24	$1.89x10^6$	N	N	N	GT
106	A4	M	44	1a	5.4*	26	48	$3.08x10^6$	N	N	N	TT
54	A4	M	44	1a	0	44	48	824830	N	N	N	GT
45	A4	F	35	1a	2	74	16	343163	N	nd	N	TT
39	A4	M	31	1b	1	72	48	$11.5x10^6$	N	Y	Y	GT
102	A4	M	30	1b	**1**	151	48	584666	N	Y	Y	GT
37	A5	M	35	1b	3	113	48	$10.2x10^6$	Y	Y	Y	TT
38	A5	M	28	1b	2^+	69	48	631606	Y	Y	Y	TT
55	A5	M	35	1a	1	76	24	398842	Y	Y	Y	TT
56	A5	F	61	1b	4	36	24	31025	Y	Y	Y	TT
40	A6	M	39	1b	2	26	48	$2.45x10^6$	N	N	N	TT
25	A6	M	43	1a	2	56	48	$8.40x10^6$	N	Y	N	TT
43	A6	M	54	1a	2	77	48	$11.65x10^6$	N	Y	Y	TT
103	A6	M	36	1b	7.3*	59	48	36678	Y	Y	Y	TT
34	B1	M	55	1a	1	41	NA	$9.03x10^6$	NA	NA	NA	TT
29	B1	F	49	1a	1	47	NA	392438	NA	NA	NA	TT
35	B2	F	55	1a	1	35	NA	831657	NA	NA	NA	GT
44	B2	F	33	1b	1	51	NA	$1.69x10^6$	NA	NA	NA	TT
46	B3	M	53	1a	1	101	NA	$2.79X10^6$	NA	NA	NA	GT
51	B3	F	43	1a	1	76	NA	$1.65x10^6$	NA	NA	NA	TT
52	B3	M	59	1a	3	46	NA	$2.55x10^6$	NA	NA	NA	GT
53	B3	M	49	1a	2	31	NA	3868	NA	NA	NA	TT

Supplementary table S2: Primers used for HCV sequencing

Primers 5'-3'	1stR PCR cycle	2ndR PCR cycle	Length	Epitope
Ext: ATCACGGCGTACGCCCAGCAGA ATCGGCGTGCCTCGTGACCA Int: AGCCTAACTGGCCGGGACAA TCCGAGGAGCCGCAAGTGCA	50°C X 30mins, 94°C X 2mins, 39 cycles of 94°C X15secs, 52°C X 30secs, 68°C X 30secs, final elongation at 68°C X 5mins	94°C X 2mins then 39 cycles of 94°C X 15secs, 55°C X30secs, 72°C X30secs then final elongation at 72°C X 2mins	250bp	CVNGVCWTV
Ext: GACAAAAACCAAGTGGAGGG GAGGACCTTCCCCAGTCC Int: CCTACGGCAAGTTCCTTGC AGCGTGRTTGTCTCAATGG	50°C X 45mins, 94°C X 2mins, 39 cycles of 94°C X 30secs, 52°C X 30secs, 68°C X 2mins, final elongation at 68°C X 10mins	94°C X 2mins then 39 cycles of 94°C X 30secs, 52°C X30secs, 72°C X30secs then final elongation at 72°C X 5mins	528bp	KLSGLGINAV, HSKKKCDEL, ATDALMTGY, TLTHPVTK
Ext: as above Int: TGTGTCACYCAGACAGTCG GGGCCCTTCTGCTTGAACTGC	As above	94°C X 2mins then 39 cycles of 94°C X 30secs, 57°C X30secs, 72°C X 1min then final elongation at 72°C X 5mins	747bp	SVVIVGRIIL QEFDEMEECASHLPY
Ext: GCTCGCYGAGCAGTTCAAGC TYGACCATGACCCGTCGC Int: GCTCGYGAGCAGTTCAAGC GGCTATYAGCCCGGTTCATCC	50°C X45mins, 94°C X 2mins, 39 cycles of 94°C X30secs, 54°C X 30secs, 68°C X 2mins, final elongation at 68°C X 10mins	94°C X 2mins then 39 cycles of 94°C X 30secs, 55°C X30secs, 72°C X 1min then final elongation at 72°C X 5mins	575bp	Geno1a: ILAGYGAGV ALVVGVVCAA
Ext: as above Int: AGGAACATGTGGAGTGGG GTTYTCTGACTCAACCCTGG	As above	94°C X 2mins then 39 cycles of 94°C X 30secs, 53°C X30secs, 72°C X 45secs then final elongation at 72°C X 5mins	570bp	Geno1a APNYSRAL FQVGLNQYLVGSQLP
Ext: GTGGAAGTGTCTCAYACG ATGTTYCCGCCCATCTCCTGCCG Int: CGARCAGGGAATGCAGCTCGC AGCCGGTTCATCCACTGC	50°C X45mins, 94°C X 2mins, 39 cycles of 94°C X30secs, 54°C X 30secs, 68°C X 2mins, final elongation at 68°C X 10mins	94°C X 2mins then 39 cycles of 94°C X 30secs, 55°C X30secs, 72°C X 45secs then final elongation at 72°C X 5mins	550bp	Geno1b ILAGYGAGV ALVVGVVCAA
Ext: as above Int: CACCTGCCCATGTGGAGC AGGGGTCGGTRAGCATG	As above	As above	430bp	Geno1b APNYSRAL FQVGLNQYLVGSQLP
Ext: CCACATCAACTCCGTGTGG TTCATCGGTTGGGGAGGAGG Int: CCACATCAACTCCGTGTGG CCACACACGAGCATGGTGC	50°C X45mins, 94°C X 2mins, 39 cycles of 94°C X30secs, 53°C X 30secs, 68°C X 2mins, final elongation at 68°C X 10mins	94°C X 2mins then 39 cycles of 94°C X 30secs, 53°C X30secs, 72°C X 45secs then final elongation at 72°C X 7mins	580bp	AYDVVSTL
Ext: GGCGACGACTTAGTCGTTATCTGTG TTCAAGCTGGTCCCTGGCTATAAGG Int: GTTATCTGTGAAAGTGCGGGGTCC GGCTATAAGGACGCTAAAGAAATGG	50°C X30mins, 94°C X 2mins, 39 cycles of 94°C X15secs, 55°C X 30secs, 68°C X 30secs, final elongation at 68°C X 5mins	94°C X 2mins then 39 cycles of 94°C X 15secs, 57°C X30secs, 72°C X 30secs then final elongation at 72°C X 2mins	288bp	LTRTDPTTPL
Ext: CCACATCAACTCCGTGTGG TTCATCGGTTGGGGAGGAGG Int: GCCTGCTACTCCATAGAACC TTCATCGGTTGGGGAGGAG	50°C X45mins, 94°C X 2mins, 39 cycles of 94°C X30secs, 53° X 30secs, 68°C X 90secs, final elongation at 68°C X 10mins	94°C X 2mins then 39 cycles of 94°C X 30secs, 55°C X30secs, 72°C X 40secs then final elongation at 72°C X 4mins	390bp	Geno 1a RPRWFMCLLLLSVG
Ext: CGCTGYTTTGACTCAACG ATTGGCCTGGAGTGTTTA Int: CYCACTTCTTCTCCATCC ATTGGCCTGGAGTGTTTA	As above	94°C X 2mins then 39 cycles of 94°C X 30secs, 51°C X30secs, 72°C X 40secs then final elongation at 72°C X 4mins	487bp	Geno 1b RPRWFMCLLLLSVG

Supplementary Table S3: Circulating HCV sequence at immunogenic T-cell epitopes

Viral sequence was determined at baseline and at any point of viral relapse (designated by 'Pt Identifier xxB'). The HCV vaccine immunogen sequence is given in the top line of each table. A dashed line below indicates that an amino acid is identical to that in the vaccine immunogen.

The epitopes to which individual patients have made a T-cell response (as measured by ex vivo IFNγ-ELISpot assay) are shown in bold **red font**. nd= not determined ? = Amino-acid not called.

Table 3: **A**) HLA-A1 epitope (TLTHPITK) **B**) HLA-B8 epitope (HSKKKCDEL) **C**) HLA-B27 epitope ARMILMTHF **D**) HLA-A2 epitopes (CVNGVCWTV, KLSGLGINAV, ALYDVVSTL, ILAGYGAGV) **E**) epitopes of unknown HLA restriction.

A)

	Pt No.	T	L	T	H	P	I	T	K
HLA-A11 +ve pts	45	**I**	**-**	**-**	**-**	**-**	**-**	**-**	**-**
	45B	I/L	-	-	-	-	-	-	-
	28	-	-	-	-	-	-	-	-
	28B	-	-	-	-	-	-	-	-
	51	-	-	-	-	-	V	-	-
HLA-A11 neg pts	43	-	-	-	-	-	V	-	-
	55	-	-	-	-	-	V	-	-
	103	-	-	-	-	-	-	-	-
	52	-	-	-	-	-	-	-	-
	106	-	-	-	-	-	V	-	-
	54	-	-	-	-	-	-	-	-
	27	-	-	-	-	-	V	-	-
	38	V	-	-	-	-	-	-	-
	53	?	?	-	-	-	-	-	-
	101	-	-	-	-	-	-	-	-
	102	-	-	-	-	-	V	-	-
	40	-	-	-	-	-	V	-	-
	40B	-	-	-	-	-	V	-	-
	48	nd							
	39	nd							
	50	nd							
	46	nd							
	36	nd							
	37	nd							
	56	nd							
	100	nd							

B)

	Pt No.	H	S	K	K	K	C	D	E	L
HLA-B8 +ve pts	45	**-**	**-**	**-**	**R**	**-**	**-**	**-**	**-**	**-**
	45B	-	-	-	R	-	-	-	-	-
	44	-	-	-	-	-	-	-	-	-
	50	-	-	-	-	-	-	-	-	-
	48	-	-	-	-	-	-	-	-	-
	46	-	-	-	-	-	-	-	-	-
	53	-	-	R	-	-	-	-	-	-
HLA-B8 neg pts	40	-	-	-	-	-	-	-	-	-
	40B	-	-	-	-	-	-	-	-	-
	25	-	-	-	-	-	-	-	-	-
	51	-	-	-	-	-	-	-	-	-
	52	-	-	-	-	-	-	-	-	-
	39	-	-	-	-	-	-	-	-	-
	103	-	-	-	-	-	-	-	-	-
	27	-	-	-	-	-	-	-	-	-
	28	-	-	-	-	-	-	-	-	-
	28B	-	-	-	-	-	-	-	-	-
	38	-	-	-	-	-	-	-	-	-
	43	-	-	-	-	-	-	-	-	-
	55	-	-	-	-	-	-	-	-	-
	54	-	-	-	-	-	-	-	-	-
	37	-	-	-	R	-	-	-	-	-
	100	-	-	-	-	-	-	-	-	-
	102	-	-	-	-	-	-	-	-	-
	56	n	d							
	101	n	d							
	36	n	d							

C)

	Pt No.	A	R	M	I	L	M	T	H	F
HLA-B27 +ve pts	54	**V**	**-**	**-**	**-**	**-**	**L**	**-**	**-**	**-**
	102	-	-	-	V	-	-	-	-	-
HLA-B27 neg pts	28	-	-	-	-	-	-	-	-	-
	28B	-	-	-	-	-	-	-	-	-
	48	-	-	-	-	-	-	-	-	-
	50	-	-	-	-	-	-	-	-	-
	39	-	-	?	-	-	-	-	-	-
	101	V	-	-	-	-	L	-	-	-
	45	-	-	-	-	-	-	-	-	-
	37	-	-	-	-	-	-	-	-	-
	38	-	-	-	-	-	-	-	-	-
	55	-	-	-	-	-	-	-	?	-
	40	-	-	-	-	-	-	-	-	-
	43	-	-	-	-	-	-	-	-	-
	103	-	-	-	V	-	-	-	-	-
	46	-	-	-	-	-	-	-	-	-
	52	-	-	-	-	-	-	-	-	-
	53	-	-	-	-	-	-	-	-	-
	51	-	-	-	-	-	?	-	-	-
	27	n	d							
	25	n	d							
	36	n	d							
	100	n	d							
	106	n	d							
	56	n	d							

D)

	Pt No.	C V N G V C W T V	K L S G L G I N A V	A L Y D V V S T L	I L A G Y G A G V
	27	- I - - - - - - -	- - V S - - L - - -	- - - - - - - K -	- - - - - - - - -
HLA-A2	28	- I - - - - - - -	- - V A - - - - - -	- - - - - - - K -	- - - - - - - - -
+ve	28B	- I - - - - - - -	- - V A - - V - - -	- - - - - - - K -	- - - - - - - - -
pts	40	- I - - - - - - -	- - V A M - V - - -	- - - - - - - K -	n d
	40B	- I - - - - - - -	- - V A M - V - - -	- - - - - - - K -	n d
	103	- I - - - - - - -	- - V A - - - - - -	- - - - - - - - -	- - - - - - - - -
	50	- I - - - - - - -	- - V A - - V - - -	- - - - - - - K -	- - - - - - - - -
	50B	n d	n d		n d
	52	- I - - - - - - -	- - V A - - V - - -	- - - - - - - K -	- - - - - - - - -
	44	- I S G A - - - -	- - V A - - V - - -	- - - - - - - - -	n d
	46	- I - - - - - - -	- - V A - - - - - -	- - - - - - - K -	- - - - - - - - -
	38	- I - - - - - - -	Q - - - - - L - - -	- - - - - - - - -	- - - - - - - - -
	36	- I - - - - - - -	n d	- - - - - - - - -	- - - - - - - - -
	43	- I - - - - - - -	- - V A - - V - - -	- - - - - - - K -	- - - - - - - - ?
	25	- I/- - - - - - - -	- - V V - - - - - -	- - - - - - - R -	- - - - - - - - -
	55	- I - - - - - - -	- - V A - - V - - -	- - - E - - - K -	n d
	37	- I - - - ? ? - -	- - - - - - L - - -	- - - - D - - - -	- - - - - - - - -
	53	- I - - - - - - -	- - V T - - V - - -	- - - - - - - - -	n d
	100	- - - - - - - - -	- - - - - - L - - -	- - - - - - - - -	- - - - - - - - -
	54	- I - - - - - - -	- - V A M - V - - -	- - - - - - - K -	n d
	56	n d	n d	n d	- - - - - - - - -
	48	- I - - - - - - -	- - V A - - V - - -	- - - - - - - K -	n d
HLA-A2	39	- I - - - - - - -	- - V A - - - - - -	- - - - - - - K -	- - - - - - - - -
-ve	45	- I - - - - - - -	- - V A - - - - - -	- - - - - - - K -	- - - - - - - - -
pts	45B	- I - - - - - - -	- - V A - - -/V - - -	- - - - - - - K -	n d
	51	- I - - - - - - -	- - V A - - V - - -	- - - - - - - K -	n d
	101	- I - - - - - - -	n d	n d	- - - - - - - - -
	102	- - - - - - - - -	- - - - - - L - - -	n d	- - - - - - - - -

E)

Pt No.	G	I	N	A	V	A	Y	Y	R	G	L	D	V	S	V
43	–	V	–	–	–	–	–	–	–	–	–	–	–	–	–
55	–	V	–	–	–	–	–	–	–	–	–	–	–	–	–
103	–	–	–	–	–	–	–	–	–	–	–	–	–	–	–
28	–	–	–	–	–	–	–	–	–	–	–	–	–	–	–
28B	–	V	–	–	–	–	–	–	–	–	–	–	–	–	–
52	–	**V**	–	–	–	–	–	–	–	–	–	–	–	–	–
106	–	V	–	–	–	–	–	–	–	–	–	–	–	–	–
25	–	–	–	–	–	–	–	–	–	–	–	–	–	–	–
45	–	I	–	–	–	–	–	–	–	–	–	–	–	–	–
045B	–	I/V	–	–	–	–	–	–	–	–	–	–	–	–	–
51	–	V	–	–	–	–	–	–	–	–	–	–	–	–	–
54	–	V	–	–	–	–	–	–	–	–	–	–	–	–	–
27	–	L	–	–	–	–	–	–	–	–	–	–	–	–	–
38	–	L	–	–	–	–	–	–	–	–	–	–	–	–	–
53	n	d													
101	n	d													
102	n	d													
40	–	V	–	–	–	–	–	–	–	–	–	–	–	–	–
040B	–	V	–	–	–	–	–	–	–	–	–	–	–	–	–
48	–	V	–	–	–	–	–	–	–	–	–	–	–	–	–
39	–	–	–	–	–	–	–	–	–	–	–	–	–	–	–
50	–	V	–	–	–	–	–	–	–	–	–	–	–	–	–
46	–	–	–	–	–	–	–	–	–	–	–	–	–	–	
36	n	d													
37	n	d													
56	n	d													
100	n	d													

Pt No.	S	V	V	I	V	G	R	I	I	L
43	C	–	–	–	–	–	–	V	V	–
55	C	–	–	–	–	–	–	–	V	–
103	C	–	–	–	–	–	–	V	V	–
28	C	–	–	–	–	–	–	V	V	–
28B	C	–	–	–	–	–	–	V	V	–
52	C	–	–	–	–	–	–	V	V	–
106	C	–	–	–	–	–	–	–	V	–
25	C	–	–	–	–	–	–	V	V	–
45	C	–	–	–	–	–	–	–	V	–
045B	C	–	–	?	–	–	–	–	V	–
51	C	–	–	–	I	–	–	V	V	–
54	C	–	–	–	–	?	–	–	V	–
27	**C**	–	–	–	–	–	–	**V**	**V**	–
38	C	–	–	–	–	–	–	V	V	–
53	C	–	–	–	–	–	?	V	V	–
101	C	–	–	–	–	–	–	–	–	–
102	C	–	–	–	–	–	–	–	V	–
40	C	–	–	–	–	–	–	–	V	–
040B	C	–	–	–	–	–	–	–	V	–
48	n	d								
39	n	d								
50	n	d								
46	n	d								
36	n	d								
37	n	d								
56	n	d								
100	n	d								

Pt No.	Q	E	F	D	E	M	E	E	C	A	S	H	L	P	Y
43	R	–	–	–	–	–	–	–	–	S	Q	–	–	–	–
55	R	–	–	–	–	–	–	–	–	S	Q	–	–	–	–
103	–	–	–	–	–	–	–	–	–	S	Q	–	–	–	–
28	R	–	–	–	–	–	–	–	–	S	Q	–	–	–	–
28B	R	–	–	–	–	–	–	–	–	S	Q	–	–	–	–
52	–	–	–	–	–	–	–	–	–	S	Q	–	–	–	–
106	R	–	–	–	–	–	–	–	–	S	Q	–	–	–	–
25	R	–	–	–	–	–	–	–	–	S	Q	–	–	–	–
45	R	–	–	–	–	–	–	–	–	S	Q	–	–	–	–
045B	R	–	–	–	–	–	–	–	–	S	Q	–	–	–	?
51	–	–	–	–	–	–	–	K	–	S	Q	–	–	–	–
54	R	–	–	–	–	–	–	–	–	S	Q	–	–	–	–
27	**R**	–	–	–	–	–	–	–	–	**S**	**Q**	–	–	–	–
38	–	–	–	–	–	–	–	M	–	del	–	–	–	–	–
53	R	–	–	–	–	–	–	–	–	S	Q	–	–	–	–
101	R	–	–	–	–	–	–	–	–	S	Q	–	–	–	–
102	R	–	–	–	–	–	–	–	–	S	Q	–			
40	R	–	–	–	–	–	–	–	–	S	Q	–	–	–	–
040B	R	–	–	–	–	–	–	–	–	S	Q	–	–	–	–
48	n	d													
39	n	d													
50	n	d													
46	n	d													
36	n	d													
37	n	d													
56	n	d													
100	n	d													

Pt No.	A	P	N	Y	S	R	A	L	W	R	V	A	A	E	E
28	–	–	–/Y	–	K	F	–	–	–	–	–	S	–	–	–
028B	S	–	–	–	K	F	–	–	–	–	–	S	–	–	–
45	–	–	–	–	K	F	–	–	–	–	–	S	–	–	–
045B	n	d													
103	–	–	–	–	–	–	–	–	–	–	–	–	–	–	–
46	–	–	–	–	T	F	–	–	–	–	–	S	–	–	–
39	–	–	–	–	**T**	**F**	–	–	–	–	–	**S**	–	–	–
38	–	–	–	–	T	F	–	–	–	–	–	–	–	–	–
55	–	–	–	–	T	F	–	–	–	–	–	S	–	–	–
56	n	d													
43	–	–	–	–	T	F	–	–	–	–	–	S	–	–	–
51	–	–	–	–	T	F	–	–	–	–	–	S	–	–	–
48	–	–	–	–	T	F	–	–	–	–	–	S	–	–	–
52	n	d													
53	–	–	–	–	K	F	–	–	–	–	–	S	–	–	–
54	–	–	–	–	T	F	–	–	–	–	–	S	–	–	–
25	–	–	–	–	T	F	–	–	–	–	–	S	–	–	–
106	–	–	–	–	T	F	–	–	–	–	–	S	–	–	–
102	n	d													
101	n	d													
36	n	d													
100	n	d													
37	n	d													
40	n	d													
040B	n	d													
27	n	d													
50	n	d													

Pt No.	L	T	R	D	P	T	T	P	L	A	R
28	–	–	–	–	–	–	–	–	–	–	–
028B	–	–	–	–	–	–	–	–	–	–	–
45	–	–	–	–	–	–	–	–	–	–	–
045B	–	–	–	–	–	–	–	–	–	–	–
103	–	–	–	–	–	–	–	–	–	–	–
46	–	–	–	–	–	–	–	–	–	–	–
39	–	–	–	–	–	–	–	–	–	–	–
38	–	–	–	–	–	–	–	–	–	–	–
55	–	–	–	–	–	**I**	–	–	–	–	–
56	–	–	–	–	–	–	–	–	–	–	–
43	–	–	–	–	–	–	–	–	–	–	–
51	–	–	–	–	–	–	–	–	–	–	–
48	–	–	–	–	–	–	–	–	–	–	–
52	–	–	–	–	–	–	–	–	–	–	–
53	–	–	–	–	–	–	–	–	–	–	–
54	–	–	–	–	–	–	–	–	–	–	–
25	–	–	–	–	–	–	–	–	–	–	–
106	nd										
102	–	–	–	–	–	–	–	–	–	–	–
101	–	–	–	–	–	–	–	–	–	–	–
36	nd										
100	nd										
37	–	–	–	–	–	–	–	–	–	–	–
40	–	–	–	–	–	–	–	–	–	–	–
040B	–	–	–	–	–	–	–	–	–	–	–
27	–	–	–	–	–	–	–	–	–	–	–
50	–	–	–	–	–	–	–	–	–	–	–

Pt No.	F	Q	V	G	L	N	Q	Y	L	V	G	S	Q	L	P
28	–	R	–	–	–	H	E	–	P	–	–	–	–	–	–
028B	–	R	–	–	–	H	E	–	P	–	–	–	–	–	–
45	–	R	–	–	–	H	E	–	P	–	–	–	–	–	–
045B	n	d													
103	–	–	–	–	–	–	–	–	V	–	–	–	–	–	–
46	–	R	–	–	–	H	–	Y	P	–	–	–	–	–	–
39	–	**R**	–	–	–	**H**	**D**	–	**P**	–	–	–	–	–	–
38	–	L	–	–	–	–	–	–	–	–	–	–	–	–	–
55	–	R	–	–	–	H	D	–	P	–	–	–	–	–	–
56	n	d													
43	–	S	–	–	–	H	D	–	P	–	–	–	–	–	–
51	–	R	–	–	–	H	–	Y	P	–	–	–	–	–	–
48	–	R	–	–	–	H	D	–	–	–	–	–	–	–	–
52	n	d													
53	–	R	–	–	–	H	E	–	O	–	–	–	–	–	–
54	–	R	–	–	–	H	D	–	P	–	–	–	–	–	–
25	–	R	–	–	–	H	D	–	P	–	–	–	–	–	–
106	–	R	–	–	–	H	D	–	P	–	–	–	–	–	–
102	n	d													
101	n	d													
36	n	d													
100	n	d													
37	n	d													
40	n	d													
040B	n	d													
27	n	d													
50	n	d													

Pt No.	A	L	V	V	G	V	V	C	A
28	–	–	–	–	–	–	–	–	–
028B	–	–	–	–	–	–	–	–	–
45	–	–	–	–	–	–	–	–	–
045B	n	d							
103	–	–	–	–	–	–	–	–	–
46	–	–	–	–	–	–	–	–	–
39	–	–	–	–	–	–	–	–	–
38	–	–	–	–	–	–	–	–	–
55	n	d							
56	–	–	–	–	–	–	–	–	–
43	–	–	–	–	–	–	–	–	–
51	n	d							
48	n	d							
52	–	–	–	–	–	–	–	–	–
53	**n**	**d**							
54	n	d							
25	–	–	–	–	–	–	–	–	–
106	–	–	–	–	–	–	–	–	–
102	–	–	–	–	–	–	–	–	–
101	–	–	–	–	–	–	–	–	–
36	–	–	–	–	G/R	–	–	–	–
100	–	–	–	–	–	–	–	–	–
37	–	–	–	–	–	–	–	–	–
40	n	d							
040B	n	d							
27	n	d							
50	n	d							
